# BMI1 regulates androgen receptor in prostate cancer independently of the polycomb repressive complex 1

**DOI:** 10.1038/s41467-018-02863-3

**Published:** 2018-02-05

**Authors:** Sen Zhu, Dongyu Zhao, Lin Yan, Weihua Jiang, Jung-Sun Kim, Bingnan Gu, Qipeng Liu, Rui Wang, Bo Xia, Jonathan C. Zhao, Gang Song, Wenyi Mi, Rong-Fu Wang, Xiaobing Shi, Hung-Ming Lam, Xuesen Dong, Jindan Yu, Kaifu Chen, Qi Cao

**Affiliations:** 1 Center for Inflammation and Epigenetics, Houston Methodist Research Institute, Houston, TX 77030 USA; 2 Center for Cardiovascular Regeneration, Houston Methodist Research Institute, Houston, TX 77030 USA; 3 Department of Cardiothoracic Surgery, Weill Cornell Medicine, Cornell University, New York, NY 10065 USA; 4 Xiangya School of Medicine, Central South University, Changsha, Hunan 410008 China; 5 Division of Hematology/Oncology, Department of Medicine, Northwestern University Feinberg School of Medicine, Chicago, IL 60611 USA; 6 Department of Urology, Peking University First Hospital, Institute of Urology, Peking University, Beijing, 100034 China; 7 Department of Epigenetics and Molecular Carcinogenesis, Division of Basic Science Research, The University of Texas MD Anderson Cancer Center, Houston, TX 77030 USA; 8 Department of Microbiology and Immunology, Weill Cornell Medicine, Cornell University, New York, NY 10065 USA; 9 Department of Urology, University of Washington, Seattle, WA 98195 USA; 10 State Key Laboratory of Quality Research in Chinese Medicine, Macau Institute for Applied Research in Medicine and Health, Macau University of Science and Technology, Macau (SAR), 999078 China; 11 Vancouver Prostate Centre, Vancouver General Hospital, Vancouver, BC V6H 3Z6 Canada; 12 Department of Urologic Sciences, University of British Columbia, Vancouver, BC V6H 3Z6 Canada; 13 Robert H. Lurie Comprehensive Cancer Center, Northwestern University Feinberg School of Medicine, Chicago, IL 60611 USA; 14 Houston Methodist Cancer Center, Houston Methodist Research Institute, Houston, TX 77030 USA

## Abstract

BMI1, a polycomb group (PcG) protein, plays a critical role in epigenetic regulation of cell differentiation and proliferation, and cancer stem cell self-renewal. BMI1 is upregulated in multiple types of cancer, including prostate cancer. As a key component of polycomb repressive complex 1 (PRC1), BMI1 exerts its oncogenic functions by enhancing the enzymatic activities of RING1B to ubiquitinate histone H2A at lysine 119 and repress gene transcription. Here, we report a PRC1-independent role of BMI1 that is critical for castration-resistant prostate cancer (CRPC) progression. BMI1 binds the androgen receptor (AR) and prevents MDM2-mediated AR protein degradation, resulting in sustained AR signaling in prostate cancer cells. More importantly, we demonstrate that targeting BMI1 effectively inhibits tumor growth of xenografts that have developed resistance to surgical castration and enzalutamide treatment. These results suggest that blocking BMI1 alone or in combination with anti-AR therapy can be more efficient to suppress prostate tumor growth.

## Abstract (web summary)

The polycomb group protein BMI1 is highly expressed in prostate cancer. Here, the authors demonstrate that BMI1 directly interacts with AR leading to increased AR signaling independently of PRC1 complex and that targeting BMI1 inhibits tumor growth of castration-resistant prostate cancer tumors.

## Introduction

Polycomb group (PcG) proteins are essential for determining cell differentiation, maintaining stem cell self-renewal, and regulating cellular memories and proliferation^1,2^. PcG proteins are known to exert their functions by forming multimeric chromatin-associated protein complexes and repressing downstream targets. The two polycomb repressive complexes (PRC1 and PRC2) are major epigenetic regulators for monoubiquitination of histone H2A at lysine 119 and methylation of histone H3 at lysine 27. The major components of mammalian PRC1 include an E3 ubiquitin ligase ring finger protein 2 (RNF2, also known as RING1B or RING2), ring finger protein 1 (RING1, also known as RING1A), chromo box proteins (CBXs), and either B lymphoma Mo-MLV insertion region 1 (BMI1, also known as PCGF4) or the paralogs of BMI1 (PCGF1, 2, 3, 5, or 6). Although BMI1 contains a ring motif, it does not have E3 ubiquitin ligase activities and has to form a complex with RING1B to ubiquitinate their substrate H2AK119 and then repress the expression levels of PRC1 targets^3^. Mammalian PRC2 consists of a histone methyltransferase, enhancer of zeste homolog 2 (EZH2), and its known binding partners, embryonic ectoderm development (EED) and suppressor of zeste 12 (SUZ12)^4^.

BMI1 is abundantly expressed in prostatic luminal epithelial cells and its levels are associated with poor prognosis of prostate cancer patients^5^. These findings suggest that BMI1 may have functions other than stem cell renewal capacity that has not been fully characterized. AR plays key roles in prostate epithelial cell differentiation and proliferation. Blocking the AR signaling is the mainstay in prostate cancer therapy, evidenced by the next-generation antiandrogens, e.g., abiraterone and enzalutamide that potently inhibit AR functions can suppress castration-resistant prostate cancer (CRPC) tumor growth. However, prostate cells can generate AR splice variants, gain-of-function mutations, or alter its functional mode independently of androgens to become therapy resistant^6,7^. Therefore, therapies that can fully block AR protein expression have been actively investigated. Since both BMI1 and AR are abundantly expressed in prostate cancer cells, whether BMI1 modulates AR protein expression and transcriptional activity remains unclear. In this study, we discovered that BMI1, independently of the PRC1 complex, binds and stabilizes AR proteins to regulate the AR pathway in prostate cancer. This discovery conceptually advances our understanding of a novel, PRC1-independent role of BMI1 in prostate cancer progression through the AR pathway. Further, our results demonstrate that BMI1 is not only a transcriptional repressor, but also a transcriptional activator through its binding partners (i.e., AR). Most importantly, here, we show that for CRPC, especially therapy (enzalutamide)-resistant CRPC, targeting BMI1 alone or in combination with anti-AR therapy effectively kills tumor cells.

## Results

### Depletion of BMI1 decreases AR protein levels and inhibits AR-signaling pathway in prostate cancer cells

**Fig. 1:**
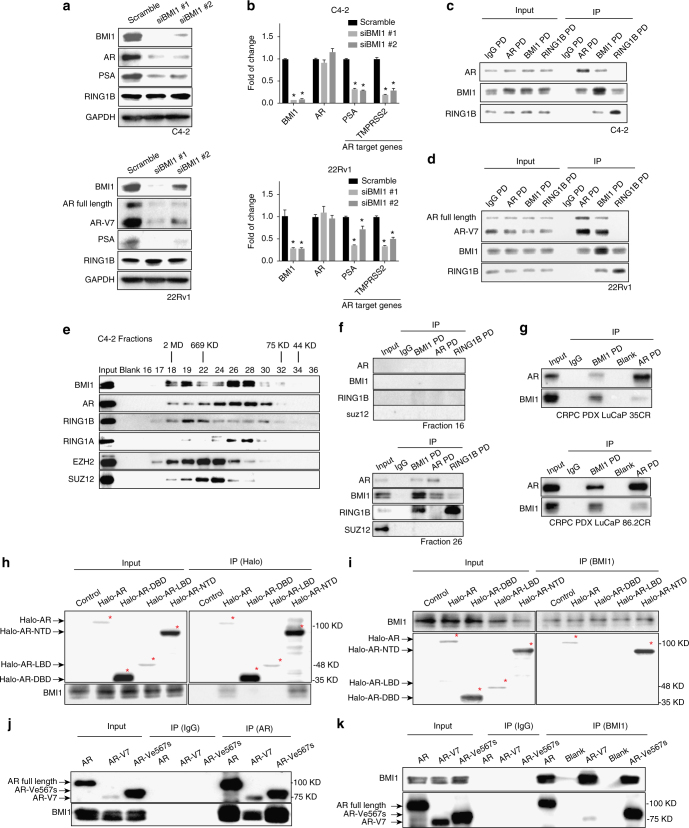
BMI1 knockdown inhibits AR signaling and BMI1 interacts with AR-NTD. **a** Protein levels following BMI1 depletion using two independent siRNA strands targeted at BMI1 (siBMI1 #1, siBMI1#2) in C4-2 cells (top panel) and 22Rv1 cells (bottom panel). **b** Transcript levels of AR and its target genes in C4-2 (top panel) and 22Rv1 cells (bottom panel) post BMI1 depletion were quantified by quantitative PCR, *P < 0.05 vs. Scramble (normalized to β-actin mRNA, mean ± SEM, *n* = 3). **c**,** d** Immunoprecipitations of C4-2 (**c**) and 22Rv1 cells (**d**) using anti-AR, anti-BMI1, or anti-RING1B antibody, rabbit IgG as control, followed by immunoblot analysis with indicated antibodies. **e** Nuclear extracts from C4-2 cells were fractionated on a Sephacryl S-300 high-resolution column. The fractions were subjected to immunoblot analysis with indicated antibodies. **f** Fractions #16 and #26 from C4-2 nuclear extracts were subjected to immunoprecipitation using anti-BMI1, anti-AR, or anti-RING1B, rabbit IgG as control, followed by immunoblot analysis using indicated antibodies. **g** PDX tissues were lysed and subjected to pull-down assay using anti-BMI1 or anti-AR antibody, rabbit IgG as control. **h** 293T cells transfected with Halo-AR (full length), Halo-DBD, Halo-LBD, Halo-NTD plasmids, or empty vector were lysed and subjected to pull-down assay using halo-tag magnetic beads or **i** anti-BMI1 antibody followed by immunoblot analysis with indicated antibodies. **j** 293T cells were transfected with full-length wild-type AR, AR-V7, and AR-V^e567s^, respectively, followed by IPs with an anti-AR antibody or **k** an anti-BMI1 antibody, rabbit IgG as control. Total cell lysates served as loading control to detect input levels of BMI1, full-length AR, and AR domains. All experiments were biologically repeated at least three times. Representative images are shown

To investigate the role of BMI1 in CRPC, we knocked down BMI1 in C4-2 cells using two distinct BMI1-specific siRNA duplexes and observed that both siRNAs decreased the expression levels of AR and prostate-specific antigen (PSA), a well-known transcriptional target of AR (Fig. 1a, upper panel). The expression levels of AR, AR variant AR-V7, and PSA were decreased by BMI1 siRNAs in another CRPC cell line, 22Rv1 (Fig. 1a, lower panel). Transcript levels were consistent with changes in protein levels of BMI1 and PSA (Fig. 1b). RNA level of TMPRSS2, another AR transcriptional target gene, was also decreased (Fig. 1b). However, the transcript levels of AR were not downregulated by BMI1 knockdown in both cell lines (Fig. 1b). Additionally, BMI1 knockdown significantly inhibited cell growth (Supplementary Fig. 1a). In order to exclude the possibility that the decrease of AR and PSA might be induced by suppressed cell growth post BMI1 knockdown, we first knocked down c-Myc or aurora kinase A (AURKA), which are well-known oncogenes and regulate cancer cell growth^8^, to dramatically suppress cell growth, and found that AR or PSA levels were not downregulated (Supplementary Fig. 1a, b). Furthermore, we treated C4-2 cells with doxorubicin, VX680, and etoposide, which are well-known compounds inhibiting cancer cell growth. As shown in Supplementary Fig. 1c, d, all these three drugs remarkably suppressed cell growth as expected. However, AR and PSA protein levels did not decrease. Taken together, cell growth arrest has no effect on the expression of AR and PSA, and AR and PSA downregulation is not due to BMI1 knockdown-induced inhibition of cell growth.

### BMI1 interacts with N-terminal domain of AR and AR variants

Since the loss of BMI1 decreased AR protein levels and inhibited AR signaling without downregulation of AR transcript levels, and our mass spectrometry analysis^9^ indicated an interaction between BMI1 and AR (Supplementary Fig. 2a), we hypothesized that BMI1 regulates the AR protein and its functions through a posttranslational modification. To test this hypothesis, we performed co-immunoprecipitation (co-IP) and western blot experiments utilizing total lysate obtained from C4-2. Immunoprecipitations of BMI1 and AR pulled down both endogenous AR and BMI1 proteins; however, RING1B did not interact with AR (Fig. 1c). We further observed that BMI1, but not RING1B, also interacted with AR-V7 as well as full-length AR proteins in 22Rv1 cells (Fig. 1d). AR-V7 lacks the ligand-binding domain and has been shown to be constitutively active in the absence of androgen^10^. As shown in Supplementary Fig. 2b, 22Rv1 cells were grown in charcoal-stripped serum medium (androgen-depleted medium) for 48 h, and co-IP using nuclear extracts illustrated the interaction between AR-V7, AR-WT, and BMI1. These results suggest that the interaction between AR (and/or variants) and BMI1 is androgen independent in the CRPC cells harboring AR variants. To further substantiate our results, we performed size-exclusion chromatography on nuclear extracts from C4-2 (Fig. 1e) and observed that AR and BMI1 proteins were coeluted in the same sized fractions. Next, we performed IP of BMI1, AR, and RING1B from different fractions as indicated. As shown in Fig. 1f, BMI1 pulled down both AR and RING1B. However, AR only pulled down BMI1 but not RING1B. On the other hand, RING1B only pulled down BMI1 but not AR. These results further confirm that AR interacts with BMI1, but not RING1B. More importantly, using two CRPC patient-derived xenograft (PDX) models^11^—LuCaP 35CR and 86.2CR—we further confirmed the interactions between AR and BMI1 in prostate cancer patient tissues (Fig. 1g).

AR is composed of three functional domains: N-terminal domain (NTD), DNA-binding domain (DBD), and ligand-binding domain (LBD)^12^. To map the interaction domain of AR and BMI1, we expressed halo-tagged AR-truncated mutants as well as full-length AR in 293T cells (Supplementary Fig. 2c). Pull-down assays with a Halo ligand or an anti-BMI1 antibody show that BMI1 interacts with AR-NTD as well as full-length AR, but not AR-DBD or AR-LBD (Fig. 1h, i). We anticipated that BMI1 would bind to all known AR variants, since each of them contains a NTD^13^. To test this, we ectopically overexpressed two major variants, AR-V7 and AR-V^e567s^ (Supplementary Fig. 2d), which have been reported to play a critical role in castration and therapy (enzalutamide) resistance in prostate cancer^6,7^. IP–IB analysis demonstrated that BMI1 indeed interacted with AR-V7 and AR-V^e567s^ without the presence of AR full length (Fig. 1j, k), which supports our hypothesis.

### BMI1 stabilizes AR protein via competitive inhibition of MDM2-mediated ubiquitination

**Fig. 2:**
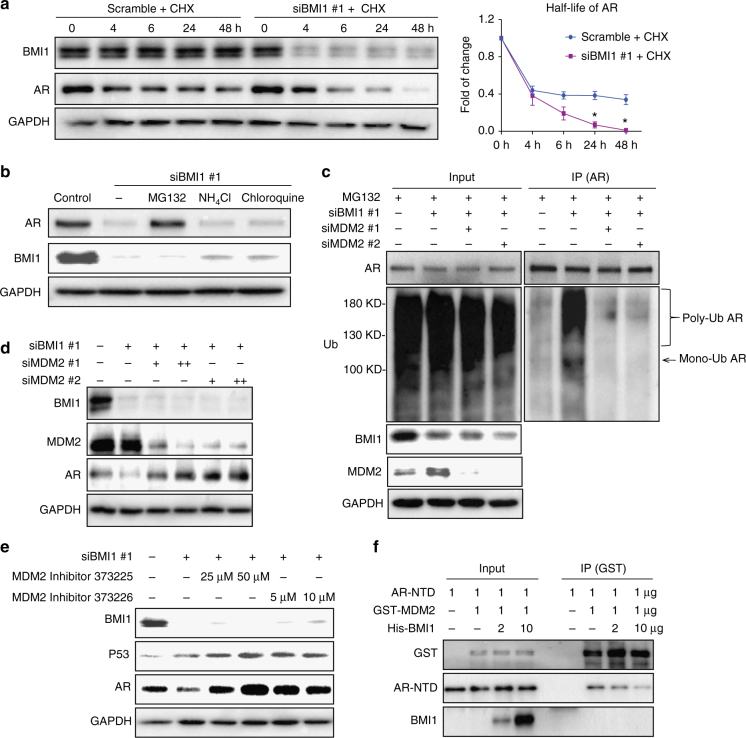
BMI1 stabilizes AR via competitive inhibition of MDM2-mediated ubiquitination. **a** C4-2 cells were transfected with siBMI1 or scramble and at 24 h post transfection, cells were treated with 10 µg ml^−1^ CHX for the time indicated. Total cell lysates were blotted for BMI1 and AR, while GAPDH served as loading control. Zero-hour time points of both treatments were immediately post transfected. *P < 0.05 vs. Scramble + CHX, mean ± SEM. **b** C4-2 cells transfected with siBMI1 were treated as labeled at 24 h post transfection for 48 h, vehicle was used as the control; 20 µM MG132, 10 mM NH_4_Cl, and 200 µM chloroquine were used. Total cell lysates were blotted for AR and BMI1, while GAPDH served as loading control. **c** C4-2 cells were transfected with siBMI1, siMDM2, or both as indicated; at 12 h post transfection, cells were treated with MG132 (20 µM) for another 36 h, and cells were then lysed and subjected to immunoprecipitation using anti-AR antibody, followed by immunoblotting with indicated antibodies. **d** BMI1 or BMI1 + MDM2 were knocked down using siRNA as indicated. Total cell lysates were immunoblotted for BMI1, AR, MDM2, and GAPDH. **e** C4-2 cells were transfected with siBMI1 or scramble. Twelve hours after transfection, MDM2 inhibitors were used to treat cells as indicated for another 36 h, followed by immunoblot analysis with indicated antibodies. GAPDH was used as a loading control. **f** Purified AR-NTD and GST-MDM2 proteins were incubated with purified His-BMI1 protein at indicated concentrations (0, 2, or 10 µg) at 4 °C for 12 h, followed by GST pull-down assay. The blots were probed with indicated antibodies. All experiments were biologically repeated at least three times. Representative images are shown

It has been reported that several E3 ubiquitin ligase proteins and/or ring finger proteins, such as RNF6 and MDM2, could bind and regulate AR protein stability or degradation^14,15^. We expected that BMI1 might play a similar role to stabilize AR proteins. To test this hypothesis, we knocked down BMI1 in C4-2 cells, treated cells with cycloheximide (CHX) to block de novo protein synthesis, and measured the half-life of AR protein. BMI1 knockdown dramatically shortens the half-life of AR (Fig. 2a). There are two major protein degradation pathways in eukaryotic cells, the ubiquitin-proteasome pathway and the lysosomal proteolysis-mediated pathway. The readily available proteasome and lysosomal inhibitors allowed a rapid analysis of the possible contributions of each pathway^16^. We found that MG132, a proteasome inhibitor^17^, could rescue BMI1 depletion-induced AR downregulation, but lysosomal inhibitors NH_4_Cl and chloroquine^18^ could not (Fig. 2b). This suggests that AR undergoes degradation in a proteasome-dependent manner, post BMI1 depletion. To further confirm that BMI1 depletion induces AR degradation, we knocked down BMI1 by siRNA and used MG132 to block AR degradation, followed by co-IP with anti-AR antibody to examine changes in AR ubiquitination levels. As shown in Fig. 2c, BMI1 depletion dramatically increased mono- and polyubiquitination of endogenous AR.

Although BMI1 has a RING finger domain, it does not have E3 ubiquitin ligase activities^3^. Because RING1B does not interact with AR (Fig. 1c), we expected that other E3 ubiquitin ligases other than RING1B were involved in BMI1 loss-mediated AR degradation. Several ubiquitin E3 ligases, including MDM2, RNF6, SIAH2, CHIP, and SPOP, have been shown to interact with AR^14,19–21^, but only MDM2 was reported to bind AR NTD and degrade AR proteins^22^. This finding led us to hypothesize that MDM2 is involved in BMI1 depletion-induced AR degradation. To test this hypothesis, we knocked down MDM2 using two independent strands of siRNA post BMI1 inhibition. Co-IP assays validated our hypothesis since MDM2 knockdown remarkably blocked BMI1 depletion-induced AR ubiquitination (Fig. 2c). To confirm MDM2’s role in this process, we measured AR protein levels after MDM2 inhibition, using MDM2 siRNAs or specific enzyme inhibitors, and found that all of them rescued BMI1 depletion-induced AR downregulation (Fig. 2d, e). This suggests that MDM2 is required for BMI1 depletion-induced AR degradation. However, p53 depletion had no effect on AR protein and signaling (Supplementary Fig. 3), excluding the possibility that MDM2 regulates AR through p53. To further demonstrate the underlying mechanism, we performed an in vitro competitive binding assay using purified AR-NTD, GST-MDM2 fusion protein, and His-tagged BMI1. As shown in Fig. 2f, BMI1 remarkably reduced the interaction between AR-NTD and MDM2 in a dose-dependent manner. These results confirm that BMI1 competitively inhibits the interaction between MDM2 and the AR-NTD to stabilize AR.

### BMI1 regulates AR-signaling pathway independently of PRC1

**Fig. 3:**
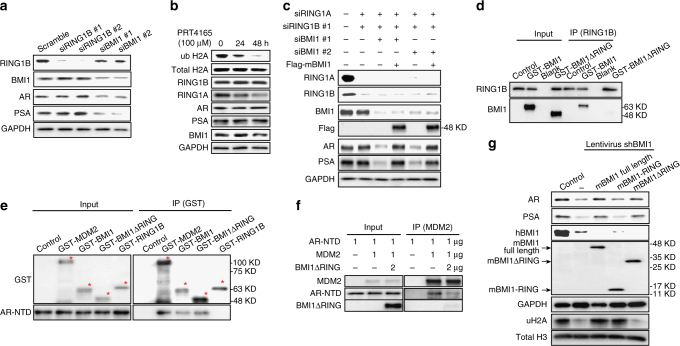
BMI1 regulates AR signaling independently of PRC1. **a** RING1B or BMI1 was depleted by siRNA in C4-2 cells. After 48 h, cells were lysed and blotted for BMI1, RING1B, AR, PSA, and GAPDH. **b** C4-2 cells were treated with PRT4165 (100 μM) for 24 or 48 h. ubH2A, RING1B, RING1A, AR, PSA, and BMI1 were tested by western blot. Total H2A and GAPDH were used as loading controls. **c** C4-2 cells were transfected with siRNA duplexes as indicated. At 24 h post transfection, cells were infected by mouse flag-BMI1 lentivirus or vector lentivirus and incubated for an additional 48 h. Cells were then lysed and immunoblot analysis was performed with indicated antibodies. This anti-BMI1 antibody only recognizes human BMI1, but not mouse BMI1. GAPDH was used as a loading control. **d** GST-BMI1 or GST-BMI1ΔRING was incubated with RING1B for 12 h followed by IPs with an anti-RING1B antibody. **e** His-AR-NTD was incubated at 4 °C with GST-MDM2, GST-BMI1, GST-BMI1ΔRING, or GST-RING1B for 12 h, followed by GST pull-down and immunoblot analysis with anti-GST and anti-AR antibodies. **f** Purified AR-NTD and MDM2 proteins were incubated with purified BMI1∆RING protein at indicated concentration (0 or 2 µg) at 4 °C for 12 h, followed by MDM2 pull-down assay. The blots were probed with anti-AR, MDM2, or anti-BMI1 antibodies. **g** C4-2 cells were infected with shBMI1 lentivirus; at 24 h post infection, cells were infected with flag-tagged mouse full-length BMI1 lentivirus, mouse BMI1-RING lentivirus, or mouse BMI1ΔRING lentivirus as indicated, and cells were lysed after another 24 h and probed by indicated antibodies. All experiments were biologically repeated at least three times. Representative images are shown

Since BMI1 is known as a key component of PRC1^23^, we wanted to determine whether regulation of AR stability by BMI1 is dependent on PRC1. Since RING1B is the core protein of PRC1, we first depleted RING1B in C4-2 cells. Surprisingly, RING1B deficiency had no effect on the protein levels of AR and its downstream protein PSA (Fig. 3a). Quantitative PCR further confirmed that there was no change at the transcript level in AR signaling post knockdown of RING1B (Supplementary Fig. 4a). Similarly, the knockdown of other PRC1 major components, including RING1A, CBX7, CBX8, or Mel-18, also showed no effect on AR signaling at the protein level (Supplementary Fig. 4b, c). However, the knockdown of RING1B markedly inhibited cell proliferation, and the knockdown of CBX7 or Mel-18 also showed significant inhibition on cell proliferation 72 h post transfection (Supplementary Fig. 4d). This result further confirms that proliferation inhibition has no effect on AR and PSA levels. Further, we observed that the previously reported PRC1 ubiquitin E3 ligase inhibitor PRT4165^24^ decreased PRC1-mediated H2A ubiquitination, but not AR or PSA levels (Fig. 3b), suggesting that inhibiting the E3 ligase activities of PRC1 does not affect AR protein levels. Therefore, we anticipated that BMI1 would regulate AR stability and AR signaling independently of PRC1 complex and its enzymatic activities. To validate this hypothesis, we knocked down both RING1B and RING1A in C4-2 cells, and did not observe any changes in AR or PSA (Fig. 3c). However, further knockdown of BMI1 in the RING1B/RING1A double-knockdown cells successfully decreased AR and PSA protein levels (Fig. 3c). In addition, overexpressing siRNA-resistant mouse BMI1 rescued BMI1 depletion-induced decreases in AR and PSA (Fig. 3c). These results strongly support our hypothesis that BMI1 regulates the AR protein and its pathway independently of PRC1.

It has been reported that BMI1 interacts with RING1B via BMI1’s conserved N-terminal RING domain^25,26^. To further investigate the PRC1-independent function of BMI1, we expressed and purified GST fusion protein containing full-length mBMI1, mBMI1-RING, and mBMI1ΔRING (Supplementary Fig. 4e). The in vitro-binding assay showed that mBMI1ΔRING loses the interaction with RING1B (Fig. 3d), while mBMI1-RING kept its strong interaction with RING1B (Supplementary Fig. 4f). mBMI1ΔRING, as well as full-length mBMI1 and MDM2, but not RING1B, interacted with AR-NTD (Fig. 3e). In addition, mBMI1ΔRING could competitively disrupt the interaction between MDM2 and AR-NTD (Fig. 3f), suggesting that BMI1ΔRING can stabilize AR. To further confirm this, we overexpressed the siRNA-resistant full-length mBMI1, mBMI1-RING domain, and mBMI1ΔRING in BMI1-depleted C4-2 and 22Rv1 cells generated using human BMI1-specific shRNA. As expected, the full-length mBMI1 and mBMI1ΔRING, but not mBMI1-RING, completely rescued BMI1 depletion-induced AR, AR-V7, and PSA downregulation (Fig. 3g and Supplementary Fig. 4g). However, mBMI1ΔRING could not rescue the BMI1 depletion-induced decrease of ubiquityl-H2AK119 in these cells (Fig. 3g). Together, this evidence strongly demonstrates that BMI1 plays a PRC1-independent role in the regulation of AR stability and signaling through its ΔRING domain, which is distinguished from its previously reported epigenetic role in self-renewing tumor-initiating cells (TICs)^27^.

**Fig. 4:**
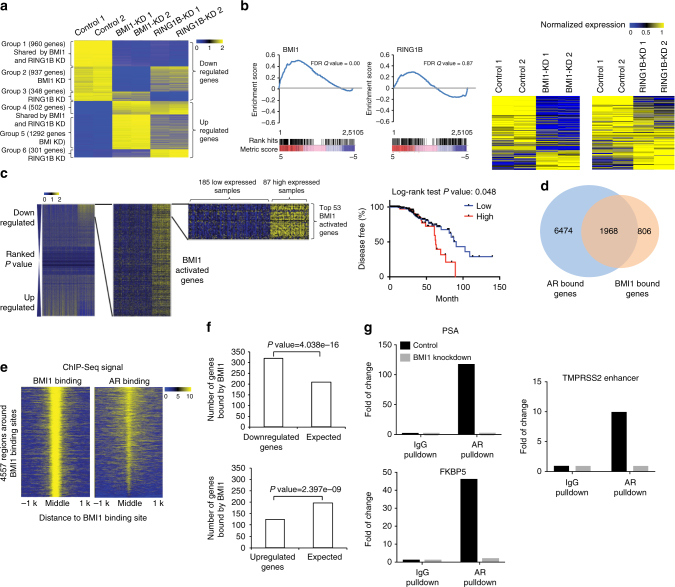
RNA-seq and ChIP-seq analysis shows that BMI1 regulates AR-signaling pathway independently of PRC1. **a** Heat maps for expression level of genes down- or upregulated by BMI1 or RING1B knockdown. **b** GSEA analysis showing enrichment level of AR-induced genes^28^ in the genes downregulated by BMI1 or RING1B knockdown, and heat maps for expression level of the AR-induced genes**. c** Kaplan–Meier (KM) analysis of prostate cancer relapse based on expression level of 53 genes that were most significantly activated by BMI1 genes. TCGA gene expression data were collected from the Cbioportal database^49,50^. Expression level of each gene in each sample was plotted in the heat map. **d** Venn diagram showing overlap of genes bound by AR and BMI1 as determined by ChIP-seq data. **e** Heat map showing the binding density of BMI1 and AR around individual binding sites of BMI1. **f** Number of genes bound by BMI1 and down- or upregulated by BMI1 knockdown. Number of genes expected by chance is also plotted. *P* value was calculated by Fisher’s exact test. **g** AR and IgG ChIP were conducted in C4-2 cells transfected with shBMI1 lentivirus or vector virus for 5 days. ChIP-qPCR was conducted using gene-specific primers

To better understand the functions of BMI1 in CRPC, we performed RNA-seq analysis using BMI1 or RING1B knockdown of C4-2 cells. We observed that 1794 genes were upregulated, while 1897 genes were downregulated by knockdown of BMI1 (Supplementary Fig. 5a), suggesting that BMI1 functions as a transcriptional activator along with its well-known transcriptional repression role in cancer. Intriguingly, while a majority (69.2%) of RING1B-regulated genes were also regulated by BMI1, only 39.6% of BMI1-regulated genes were also regulated by RING1B, indicating that BMI1 plays an additional role separate from its canonical functions as a component of PRC1. Gene expression-profiling analysis further revealed that genes regulated by BMI1 and/or RING1B could be clustered into six groups: downregulated (group 1) or upregulated (group 4) by both BMI1 and RING1B knockdown; downregulated (group 2) or upregulated (group 5) by BMI1, but not RING1B knockdown; and downregulated (group 3) or upregulated (group 6) by RING1B, but not BMI1 knockdown (Fig. 4a). We analyzed 113 AR-regulated genes derived from cell lines, human prostate cancer, and castration-resistant prostate cancer tissues (Supplementary Table 4), and found that their expression levels were significantly dysregulated by BMI1 knockdown, but not by RING1B knockdown (Supplementary Fig. 5b). Gene set enrichment analysis (GSEA) using androgen-induced genes^28^ further confirmed that AR-activated genes were significantly enriched in genes downregulated in BMI1 knockdown, but not in RING1B knockdown (Fig. 4b). Gene expression heat maps also confirmed that AR-induced genes were significantly downregulated by BMI1 knockdown, but not by RING1B knockdown (Fig. 4b). Intriguingly, KEGG pathway enrichment analysis revealed that BMI1-activated genes, but not BMI1-repressed genes, were associated with protein lysine degradation, prostate cancer, and several other cancers (Supplementary Fig. 5c). Importantly, survival analysis of two prostate cancer gene expression data sets (see “Methods” section) revealed that higher expression levels of BMI1-activated genes (those downregulated by BMI1 knockdown) were significantly associated with poorer disease-free (Fig. 4c) and poorer overall survival (Supplementary Fig. 5d). In addition, patients with mutations or copy number alterations of these BMI1-activated genes also had shorter disease-free time, compared to patients without these genomic alterations (Supplementary Fig. 5e).

To further investigate how BMI1 plays a role in AR signaling, we performed ChIP-seq analysis using anti-AR and anti-BMI1 antibodies. Our analysis revealed that AR and BMI1 were recruited to 8442 and 2774 genes, respectively. Intriguingly, AR was recruited to 70.9% (2.11-fold larger than random expectation) of BMI1-occupied genes (Fig. 4d) and they shared the binding sites on most of these genes (Fig. 4e), suggesting that BMI1 may directly regulate AR targets. In addition, the genes bound by BMI1 significantly overlapped with BMI1-activated genes, but not with BMI1-repressed genes, suggesting that the binding of BMI1 on chromatin is more likely to activate transcription (Fig. 4f). We also examined the expression levels of several AR-activated genes using qPCR and found that BMI1 depletion decreased their mRNA level (Supplementary Fig. 5f), suggesting that AR-activated genes are also activated by BMI1. ChIP-qPCR analysis further confirmed that BMI1 knockdown (Supplementary Fig. 5g) decreased the enrichments of AR in upstream regions of well-known AR target genes (Fig. 4g) and decreased the enrichments of BMI1 in BMI1 target genes (Supplementary Fig. 5h).

### BMI1-specific antagonist inhibits CRPC and drug-resistant CRPC progression in vivo

**Fig. 5:**
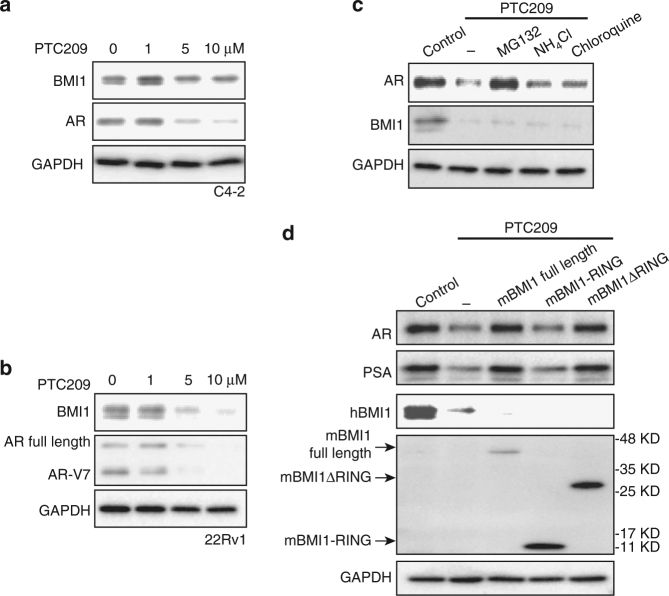
BMI1-specific antagonist inhibits AR-signaling pathway. C4-2 (**a**) or 22Rv1 (**b**) was treated with PTC209 in indicated concentration for 48 h, BMI1 and AR were tested by western blot, and GAPDH served as loading control. **c** C4-2 cells were treated for 48 h as labeled, vehicle as control, and the concentration of each drug was PTC209 5 µM, MG132 20 µM, NH_4_Cl 10 mM, and chloroquine 200 µM. Total cell lysates were blotted for AR and BMI1, while GAPDH served as loading control. **d** C4-2 cells were treated with PTC209 (5 µM), and after 24 h, cells were infected with BMI1 lentivirus, BMI1-RING lentivirus, or BMI1ΔRING lentivirus as indicated in the presence of PTC209, and cells were lysed after another 24 h and probed by indicated antibodies. All experiments were biologically repeated at least three times. Representative images are shown

**Fig. 6:**
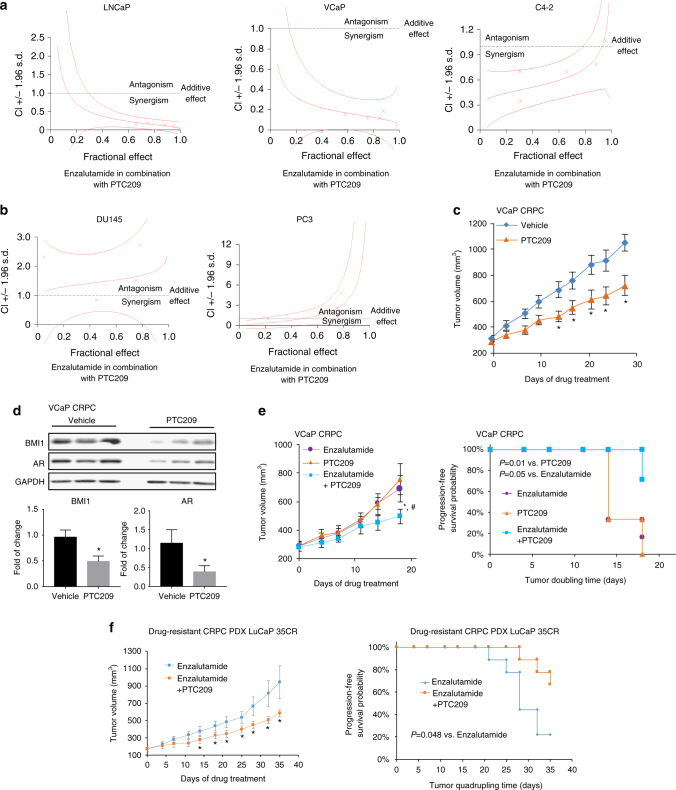
BMI1 inhibition delays CRPC progression in vivo. **a**, **b** Representation of combination index plot of enzalutamide combined with PTC209 in **a** AR-positive cells and **b** AR-negative cells. Doses below the dotted line represent the synergistic effect and doses above the dotted line represent the antagonistic effect. **c** Castration-resistant VCaP xenograft mouse models were generated as described in “Methods” section. Castrated mice carrying CRPC xenograft received vehicle or PTC209 (60 mg kg^−1^ per day) 5 days per week (*n* = 12 per group). Caliper measurements were taken every 4 days to obtain tumor volume. Mean tumor volume ± SEM, *P < 0.05 vs. Vehicle. **d** Tumor tissues were lysed and blotted for BMI1, AR, and GAPDH. The upper panel shows the representative western blot. Protein levels were quantified and normalized against GAPDH (lower panel), *P < 0.05 vs. Vehicle (mean ± SEM, *n* = 6). **e** Castrated mice carrying CRPC xenograft received enzalutamide (10 mg kg^−1^ per day), PTC209 (60 mg kg^−1^ per day), or PTC209 (60 mg kg^−1^ per day) + enzalutamide (10 mg kg^−1^ per day) 5 days per week (*n* = 6 per group). Caliper measurements were taken every 4 days to obtain tumor volume (**e**, left panel). Mean tumor volume ± SEM; **e**, right panel, Kaplan–Meier survival plot compares the progression-free survival; *P < 0.05, PTC209 + enzalutamide vs. enzalutamide; ^#^*P* < 0.05, PTC209 + enzalutamide vs. PTC209. **f** Castrated mice carrying LuCaP 35CR, an enzalutamide-resistant and abiraterone-resistant PDX model, received enzalutamide (10 mg kg^−1^ per day) or PTC209 (60 mg kg^−1^ per day) + enzalutamide (10 mg kg^−1^ per day), 5 days per week (*n* = 9 per group). Caliper measurements were taken every 4 days to obtain tumor volume. **f**, left panel, mean tumor volume ± SEM; *P < 0.05. **f**, right panel, Kaplan–Meier survival plot compared with the progression-free survival; *P = 0.048

It has been reported that the newly developed BMI1 inhibitor PTC209 effectively decreased colon, lung, and prostate cancer stem cell-mediated tumor growth^27,29,30^. Similar to BMI1 siRNA knockdown, PTC209 treatment also decreased AR, AR-V7, as well as BMI1 expression levels (Fig. 5a, b). PTC209 treatment also shortened AR half-life in CRPC cells (Supplementary Fig. 6), and proteasome inhibitor MG132 abolished the effect of PTC209 on AR protein levels (Fig. 5c). Furthermore, full-length BMI1 and BMI1ΔRING, but not BMI1-RING, rescued PTC209-induced AR and PSA downregulation (Fig. 5d). These findings are consistent with our previous results, confirming that targeting BMI1 inhibits AR signaling. To test if PTC209 could effectively inhibit the growth of AR-positive prostate cancer and CRPC, we first examined the effect of PTC209 on cell proliferation in AR-positive PCa cell lines LNCaP, VCaP, C4-2, and 22Rv1, and observed a low IC50 (around 0.6 µM) (Supplementary Fig. 7). We further observed that PTC209 and enzalutamide (an FDA-approved AR antagonist treatment for metastatic CRPC)^31^ synergistically inhibited cell proliferation of LNCaP, VCaP, and C4-2 (Fig. 6a). As expected, PTC209 and enzalutamide had no synergistic effect on the growth of AR-negative PCa cell lines DU145 and PC3 (Fig. 6b).

To assess the therapeutic effect of BMI1 in CRPC, we utilized several mouse xenograft models. Since our data strongly support that BMI1 regulates AR signaling in CRPC cells, and treatment of CRPC is still limited, we first used a castration-resistant VCaP xenograft mouse model^32^ to evaluate the therapeutic potential of BMI1 inhibition in CRPC, and PTC209 treatment significantly reduced tumor growth compared to vehicle control treatment (Fig. 6c). Mice bearing castration-resistant tumors demonstrated markedly faster progression of the disease compared with the treatment group, and PTC209 treatment had no effect on body weight (Supplementary Fig. 8). Additionally, the protein levels of BMI1 and AR were remarkably downregulated in PTC209-treated CRPC tumors when compared to those of the control group (Fig. 6d). Next, we evaluated a potential synergistic effect of combination treatment with PTC209 and enzalutamide on CRPC tumor growth. While PTC209 showed similar inhibition potential as enzalutamide, combinatorial treatment of PTC209 and enzalutamide showed significantly better outcomes compared to each treatment alone (Fig. 6e and Supplementary Fig. 9a) and had no effect on body weight (Supplementary Fig. 9b).

The rapid development of therapy resistance in prostate cancer patients subjected to enzalutamide treatment is becoming a major clinical challenge^33^. One of the important mechanisms of enzalutamide resistance is the increased expression of AR variants lacking the ligand-binding domain, the best characterized of which is AR-V7^6,7^. Using LuCaP 35CR^11^, an enzalutamide-resistant and abiraterone-resistant CRPC PDX model, we found that combinatorial treatment of PTC209 and enzalutamide significantly improves the clinical outcomes when compared with those utilizing enzalutamide alone (Fig. 6f and Supplementary Fig. 10a). And there was no difference in body weight between these two groups (Supplementary Fig. 10b). To further confirm this result, we constructed enzalutamide-resistant xenograft model: 20 castrated male SCID mice carrying xenografts of 22Rv1 tumors were treated with enzalutamide for 4 weeks, and then sections of the biggest tumor (~1500 mm^3^) among these mice were transplanted to the next few passages of castrated male SCID mice treated with enzalutamide to maintain the enzalutamide resistance. These mice were randomly divided into two groups when the tumors reached 100 mm^3^. One group was treated with PTC209 plus enzalutamide for 21 days, whereas the other group was treated with enzalutamide alone to serve as a control (Supplementary Fig. 10c). Compared to the control, PTC209 treatment significantly decreased enzalutamide-resistant CRPC tumor growth (Supplementary Fig. 10d, e). There was no difference in body weight between these two groups (Supplementary Fig. 10f). All these results strongly suggest that BMI1 is a novel therapeutic target for CRPC, especially for clinically enzalutamide-resistant CRPC.

## Discussion

**Fig. 7:**
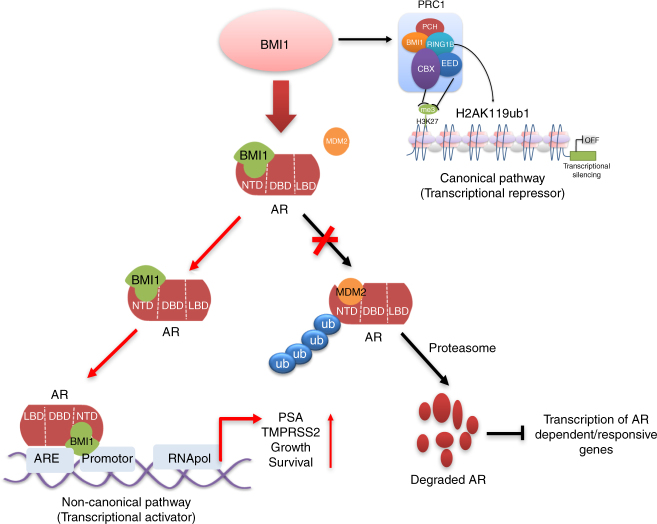
Model for the regulation of BMI1 on androgen receptor independently of PRC1. Besides its PRC1-dependent role (canonical pathway) as a transcriptional repressor, BMI1 also has a PRC1-independent role (noncanonical pathway) as a transcriptional activator: BMI1 stabilizes AR and activates AR-signaling pathway via competitively inhibiting MDM2-induced ubiquitination and degradation of AR in PCa

Recently, many significant advances have been made toward understanding how BMI1 plays a role in development, differentiation, stemness regulation, apoptosis, autophagy, cell fate reprogramming, epithelial–mesenchymal transition (EMT), and neoplastic progression^34–36^. The functions of BMI1 in stem cells and cancer stem cells (tumor-initiating cells) have also been extensively investigated^37^. The leading dogma suggests that BMI1 is a key component of PRC1 and a transcriptional repressor (Fig. 7). The newly developed BMI1 inhibitor PTC209 has efficacy in colon and lung cancer by targeting cancer stem cells^30^ and has been shown to inhibit prostate cancer stem cell tumor growth, which is usually AR negative^27^.

In this study, we made the unexpected observation that AR protein levels, but not transcript levels, as well as the protein and transcript levels of AR-activated targets PSA and TMPRSS2, were decreased by depletion of BMI1 in AR-positive prostate cancer cells. These results suggest that BMI1 loss-mediated AR downregulation is through a novel BMI1-mediated protein posttranslational function and not through its canonical role as a transcriptional repressor. We also demonstrated that BMI1 directly binds to full-length AR and AR variants through NTD in vitro and in vivo, and loss of BMI1 induces proteasome-mediated full-length AR and AR variants protein degradation.

Here, we show that BMI1 disassociates the interaction between AR and MDM2, thereby inhibiting MDM2-mediated AR ubiquitination and degradation. Intriguingly, the knockdown of key PRC1 proteins RING1B or RING1A, the H2A E3 ubiquitin ligases, or treatment with the PRC1 ubiquitin E3 ligase inhibitor PRT4165 have no effect on AR or AR target expression levels. However, a further knockdown of BMI1 in RING1A^low^/RING1B^low^ CRPC cells decreases AR protein and targets, and overexpressing siRNA-resistant BMI1 could completely restore the AR protein and its functions. To further investigate the PRC1-independent BMI1 function in prostate cancer, we demonstrated that the BMI1-truncated mutant, BMI1ΔRING, which does not contain the RING domain and cannot interact with RING1B, still directly interacts with AR NTD. Overexpression of BMI1ΔRING completely rescues the BMI1 loss-mediated decrease of AR protein and its targets, but could not rescue BMI1 loss-mediated decrease of H2A ubiquitination (which needs PRC1 activities). Importantly, our studies further demonstrate that targeting BMI1 by PTC209 significantly inhibits CRPC tumor growth, and combinational targeting of BMI1 and AR achieves better efficacy than single agents alone.

Inherent or acquired resistance to contemporary AR inhibition, such as enzalutamide, remains a major clinical obstacle. Based on our current understanding, there are three general mechanisms: restored AR signaling (AR-LBD mutation and splice variants), bypass of AR, and complete AR independence (lineage plasticity)^38^. Two recent studies demonstrated that Rb1, Trp53, EZH2, and SOX2 were involved in the prostate cancer lineage plasticity^39,40^. Here, we show that targeting BMI1 to regulate AR and its degradation of variants is effective for enzalutamide-resistant CRPC. This suggests that BMI1 may be another novel therapeutic target for enzalutamide-resistant CRPC patients.

Recent studies indicated that another PcG protein, EZH2, the core protein of PRC2 complex, might play a PRC2-independent role in CRPC^41^. However, the mechanism by which EZH2 regulates AR pathway is yet unclear. Here, we demonstrate that, unlike the canonical transcriptional repressor and its functions in cancer stem cells, BMI1 performs its noncanonical function as a transcriptional activator and an AR cofactor. Here, we show that BMI1 directly binds to AR protein and regulates AR targets in a PRC1-independent manner in AR-positive CRPC (Fig. 7), and this is the first time that BMI1 non-epigenetic and non-stemness functions have been reported.

## Methods

### Drug treatments and antibodies

Drug concentrations (unless otherwise indicated) were PTC209 (0, 1, 5, and 10 µM, HY-15888, MedChem Express), enzalutamide (IN034, Dieckmann), MDM2 inhibitor 373225 (25 µM, 50 µM, Millipore), MDM2 inhibitor 373226 (5 µM, 10 µM, Millipore), PRT4165 (100 µM 5047, TOCRIS), MG132 (CAS 133407-82-6 Calbiochem), ammonium chloride (NH_4_Cl, 12125-02-9, Sigma), chloroquine diphosphate salt (C6628, Sigma), and cycloheximide (CHX, C7698, Sigma), and all were commercially obtained. Serial dilutions of enzalutamide were made using a vehicle of 0.5% CMC (C9481, Sigma) and 0.25% Tween-80 (P8074, Sigma). PTC209 was made using a vehicle of 14% DMSO, 36% polyethylene glycol 400, and 50% polypropylene glycol 400. Antibodies used for immunoblot assays are listed in Supplementary Table 1. Protein lysates were prepared in SDS-sample buffer (4× reducing, BP-110R, Boston BioProducts). The secondary antibodies were Clean-Blot IP Detection Reagent (HRP, 21230, Thermo Scientific), goat anti-mouse IgG (H+L)-HRP (SA001-500, GenDEPOT), or goat anti-rabbit IgG (H+L)-HRP (SA002-500, GenDEPOT). Purified 6×His AR-NTD protein was bought from Ray Bioteck (RB-14-0003P). Purified His-BMI1 was bought from ORIGENE (TP760041). The Glutathione Sepharose 4B (GST beads) (17-0756-01, GE Healthcare) was used for in vitro IP. RING1B siRNA (#1, s12067, or #2 s12068), MDM2 siRNA (#1, 58628 or #2, 58629), or BMI1  siRNA (#1 s2015, #2 s2016) were all purchased from Ambion. Lipofectamine 2000 reagent (1815561, Invitrogen) and Opti-Mem (1774114, Gibco) were used in transfection. One shRNA construct targeting BMI1 (TRCN0000020156, NM_005180.5-1061s1c1) used to construct lentivirus to silence BMI1  expression was obtained from Sigma-Aldrich.

### Cell culture

LNCaP, VCaP, and 22Rv1 were purchased from ATCC. C4-2 cells were provided by Dr. Arul M. Chinnaiyan. All cells used in this study were within 20 passages after receipt. LNCaP, C4-2, and 22Rv1 were cultured in 5% CO_2_ and maintained in vitro in RPMI 1640 medium supplemented with 10% FBS, 2 mmol l^−1^ glutamines, 100 units ml^−1^ penicillin, and 100 mg ml^−1^ streptomycin. VCaP cells were maintained in Dulbecco’s modified Eagle’s medium with high glucose (HyClone) containing 10% bovine growth serum (BGS), 1% l-glutamine, and 1% penicillin and streptomycin and cultured at 37 °C in 5% CO_2_. These cell lines were recently authenticated by the University of Arizona Genetics Core using short tandem repeat (STR) profiling. Cell lines were mycoplasma negative during routine tests.

### Transfection

All the siRNA sequences were purchased from Thermo Scientific, RING1B siRNA: #1 s12067, #2 s12068; MDM2 siRNA: #1 s58628 or #2 s58629 or BMI1  siRNA (#1 s2015, #2 s2016). Briefly, 3×10^5^ cells were plated in six-well plates, grown without antibiotics to 80% confluence, and then transfected with siRNA sequences or their corresponding mock sequences using a Lipofectamine 2000 kit (Invitrogen, Cat. 11668-019) with the procedure provided by the manufacturer.

### Lentiviral constructs

Lentivirus was packaged by co-transfection of constructs with third-generation packaging plasmids pMD2.G, pRRE, and pRSV/REV with Fugene HD (Roche) into 10-cm plates with HEK293T cells. After the first 24 h of transfection (2 μg of MDLG, 1 μg of VSVG, 1 μg of Rev, and 3 μg of target plasmid), the medium was changed to DMEM, and the supernatants 72 and 96 h after transfection were pooled, filtered through a 0.45-µm filter, and stored at −80 °C. Pellets were resuspended in Opti-MEM (Gibco) and titers were determined by p24 assay, in addition to functional titration to determine an MOI of 1 for each initial batch of virus. Expression was verified by western blotting.

### Immunoprecipitation and western blot

Whole-cell lysate IP was performed by lysing cells in the NP-40 lysis buffer (2×) (100 mM Tris-HCl, pH 8.0, 300 mM NaCl, 10 mM EDTA, 2% NP-40, and N1200-050, GenDEPOT, for IP) or Pierce RIPA Buffer (89901, Thermo Scientific, for WB) with a protease and phosphatase inhibitor (1861280, Thermo). The lysate was kept on ice for 20 min and sonicated for 2 s on and 2 s off for 40 s and insoluble material was removed by centrifugation. Lysates were precleared using Dynabeads protein A (10002D, Invitrogen) or protein G (10004D, Invitrogen). Antibodies were added to lysates and incubated at 4 °C overnight. The immune complexes were collected using Dynabeads protein A (10002D, Invitrogen) or protein G (10004D, Invitrogen), and beads were washed three times extensively with the lysis buffer. To denature proteins, lysates were added to 2× reducing SDS-sample buffer or 4× reducing buffer (BP-110R, Boston BioProducts) and heated to 95 °C for 10 min. Protein levels were assessed by standard SDS–polyacrylamide gel electrophoresis and transferred to PVDF membranes (162-0177, BIO-RAD). Images were captured using the ChemiDoc XRS+ Molecular Imager system (BIO-RAD). Antibodies used in western blotting were described above. Blots were incubated overnight with primary antibodies at 4 °C, followed by detection with Clean-Blot IP Detection Reagent (HRP) (21230, Thermo Scientific), goat anti-mouse IgG (H+L)-HRP (SA001-500, GenDEPOT), or goat anti-rabbit IgG (H+L)-HRP (SA002-500, GenDEPOT) secondary antibody.

AR ubiquitination was detected under immunoprecipitation as previously described^14^. The cell lysates were added to 2% SDS and heated to 95 °C for 10 min. The lysates were then diluted using lysis buffer to reduce SDS concentration to 0.2% before anti-AR or anti-Ub antibody was added. Immunoblotting was performed as described above.

As with in vitro immunoprecipitation, purified tagged proteins (dose as indicated) were added to chilled PBS (1 ml) with a protease and phosphatase inhibitor. A 50-μl aliquot was removed, rotated at 4 °C overnight, and used as the input. Proteins were denatured using 2× loading buffer and boiled for 5 min. The immune complexes were then washed three times with NP-40 lysis buffer, collected using the corresponding beads, and rotated at 4 °C for 2 h. The beads were then washed three times extensively with NP-40 lysis buffer. To denature proteins, the lysates were added to 2× reducing SDS-sample buffer and boiled for 10 min. Immunoblotting was performed as described above (whole-cell lysate IP). Unprocessed blot images for western blot analysis are shown in Supplementary Figs. 11–13.

### Size-exclusion chromatography

C4-2 nuclear extracts were obtained using NE-PER nuclear extraction kit (Thermo Scientific), and 5 mg of nuclear protein was concentrated to 3000 μl using Microcon centrifugal filter (Millipore)^12^. This was then applied to a Sephacryl S-300 HR column (GE Healthcare) precalibrated using the Gel Filtration HMW Calibration Kit (GE Healthcare). A volume of 2000 μl of elute was collected for each fraction at a flow rate of 0.25 ml min^−1^, and eluted fractions were subjected to western blotting and co-IP.

### RNA isolation and RT-PCR from cell lines

Total RNA was isolated from cells to generate cDNA using the RNA MiniPrep kit (Direct-zol, R2052, ZYMO Research) and amfiRivert cDNA Synthesis Platinum Master Mix (R5600-100, GenDEPOT). Each cDNA sample was amplified using Power SYBR Green PCR Master Mix (4367659, Applied Biosystem) on the QuantStudio 6 Flex Real-time PCR System (403115082, GE Healthcare). Briefly, the reaction conditions consisted of 2 μl of cDNA and 0.2 μM primers in a 10-μl final volume of super mix. Each cycle consisted of denaturation at 95 °C for 15 s, annealing at 58.5 °C for 5 s, and extension at 72 °C for 10 s, respectively. β-actin was used as an endogenous control to normalize each sample. The experiment was performed in triplicate with three independent experiments. The primers are listed in Supplementary Table 2.

### Fusion protein induction and purification

*Escherichia coli* strain DH5α that harbors the expression plasmid for GST ± YKR079C or GST ± ELAC1 was incubated at 37 °C in 250 ml of LB medium containing 50 mg ml^−1^ ampicillin until the A600 of the culture reached 0.6^42^. At this point, the fusion protein was induced by adding IPTG (500 mM). After further incubation at 37 °C for 4 h, the cells were harvested by centrifugation. Cell pellets were resuspended in 10 ml of lysis buffer (50 mM Tris ± HCl, pH 7.6, 500 mM NaCl, 10% glycerol, 1 mM dithiothreitol (DTT), 23 mM AEBSF, 100 mM EDTA, 2 mM bestatin, 0.3 mM E-64, and 0.3 mM pepstatin A). The cells were then sonicated and centrifuged at 100,000 × *g* for 1 h. The cleared lysate was incubated with 0.5 ml of glutathione ± sepharose beads at 4 °C for 12 h. After exhaustive washing, the retained proteins were eluted from the beads with 0.1 ml of buffer (50 mM Tris ± HCl, pH 7.6, 10% glycerol) containing 20 mM glutathione. All the purification steps were carried out at 4 °C.

### RNA-sequencing analysis

The RNA-seq reads were mapped to the human reference genome version hg19 using TopHat (version 2.1.0)^43^. We downloaded the human reference gene set (Refseq gene) from https://www.ncbi.nlm.nih.gov/refseq/rsg/. The number of fragments that originated from each gene in each sample is estimated using cuffdiff in the software Cufflinks (version 2.2.1)^44^ with classic-fpkm normalization method. The differentially expressed genes based on read counts are inferred using edgeR R package v3.12 after library-size factor normalization. MEV version 4.8.1^45^ was used to plot heat maps.

### Function enrichment analysis

DAVID (version 6.8)^46^ was employed for KEGG pathway analysis. Each pathway returned by DAVID with a *P* value smaller than 1 × 10^−2^ was defined as significantly enriched. GSEA (version 2.2.0)^47,48^ was used to analyze the enrichment level of AR-induced genes collected from literature^28^.

### Survival and disease-free analysis

The Kaplan–Meier survival analysis was carried out using Cbioportal (version 1.4.3)^49,50^, PROGgene (version 2)^51^, or GraphPad Prism (version 7.0b). Statistical significance of the difference between the survival curves for different groups of patients was assessed using log-rank test.

### ChIP-seq analyses

All ChIP-seq sequencing reads were mapped to version hg19 of the human genome using Bowtie (version 1.1.2)^52^. The Dregion function in DANPOS (version 2.2.3)^53^ was used to calculate read density and define enrichment peaks. Briefly, we extended each read at the 3′ end to be 200-bp long and then calculated read density as the number of reads covering each base pair in the genome. For each sample, the total number of mapped reads was normalized to 25 million. For AR and BMI1 data, we used Poisson test *P* value 1 × 10^−35^ for read density cutoff to define seed peaks. We then used Poisson test *P* value 1 × 10^−15^ for read density cutoff to extend each seed peak. DANPOS subtracted input background signal from the ChIP signal. To further reduce the input background effect, we removed any ChIP-seq peaks that overlap with a peak in the input data. For input data, we used Poisson test *P* value 1 × 10^−15^ for read density cutoff to define seed peaks. We then used Poisson test *P* value 1 × 10^−10^ for read density cutoff to extend each seed peak. The selector function in DANPOS (version 2.2.3) was used to map the peak regions to TSSs. The overlap function in DANPOS (version 2.2.3) was used to calculate the number of overlapped peak regions.

### Chromatin immunoprecipitation

Chromatin immunoprecipitation (ChIP) was performed using the ChIP Assay kit (Millipore, Cat. 17-295) with the procedure provided by the manufacturer. For PCR analysis of enrichment of target gene promoters, 2 μl each of input DNA, AR-enriched, or IgG-enriched DNA were subjected to PCR using Platinum PCR Super mix (Invitrogen) and primers specific for target gene promoters or enhancers (Supplementary Table 3).

### Cell growth assay

Cells were seeded in 96-well plates and treated with the two single drugs, and the combination of both at concentration gradients for 96 h. Bioluminescence was measured to quantify cell survival by using CellTiter-Glo^®^ Luminescent Cell Viability Assay Kit and was read on Synergy 2 Multi-Mode Reader. The bioluminescence results were quantitated into cell viability. And drug effects (one-cell viability) were analyzed by Calcusyn (Biosoft, Ferguson, MO). Combination index (CI) values were then calculated to determine the synergy of the two drugs. Optimal concentration ratio of the two drugs was selected by CI values that showed best synergistic effects.

### Murine prostate tumor xenograft model

CB17SCID mice were purchased from Charles River. Animal care and use conditions were followed in accordance with institutional and National Institutes of Health protocols and guidelines, and all studies were approved by Houston Methodist Institution Animal Care and Use Committee. Tumor xenograft model was induced as previously described^32^. Mice were anesthetized using 2% isoflurane (inhalation) and 2 × 10^6^ VCaP or 22RV1 prostate cancer cells suspended in 100 μl of PBS with 50% Matrigel were implanted subcutaneously into the dorsal flank on both sides of the mice. Tumor volumes were measured by length (*a*), width (*b*), and calculated as tumor volume = MIN(*a*)^2^ × MAX(*b*)/2. For VCaP castration-resistant prostate tumor model, VCaP tumor-bearing mice were castrated when the tumors were approximately 200–300 mm^3^ in size and once tumors started to grow back, mice were randomized and treated with vehicle or enzalutamide  (10 mg kg^−1^) and/or PTC209 (60 mg kg^−1^) daily (5 days per week), and terminated about 28 days later. Loss of body weight during the course of the study was also monitored. For the antitumor agent combination experiment, 22RV1 castration-resistant prostate tumors were allowed to grow after castration for 3–4 weeks (to tumor volume 100 mm^3^), treated with enzalutamide  (10 mg kg^−1^, oral gavage daily) for about 28 days, and then the biggest tumor (~1500 mm^3^) was transplanted to nude mice. The mice were kept until tumor volume reached 100 mm^3^, and then randomized to two groups, treated with enzalutamide (10 mg kg^−1^, gavage) or enzalutamide combined with PTC209 (60 mg kg^−1^, S.C.) daily (5 days per week), and terminated about 28 days later.

The LuCaP 35CR and LuCaP 86.2CR patient-derived xenografts (PDX) were kindly provided by Eva Corey (University of Washington). Castrated CB17SCID mice were implanted subcutaneously with tumor bits of LuCaP 35CR. When the tumors were approximately 200 mm^3^ in size, mice were randomized and treated with enzalutamide (10 mg kg^−1^ per day) or PTC209 (60 mg kg^−1^ per day) + enzalutamide (10 mg kg^−1^ per day) (5 days per week), and terminated about 35 days later. Caliper measurements were taken every 4 days and loss of body weight during the course of the study was also carefully monitored.

### Statistical analysis

No statistical method was used to predetermine sample size. Mice were assigned at random to treatment groups and, where possible, mixed among cages. There were no inclusion or exclusion criteria. Whenever possible, the investigators were blinded to group allocation during the experiments and when assessing outcomes. Experiments were repeated two to three times. Data were analyzed using Prism 5.0 software (GraphPad) and presented as means ± SEM. The *P* values were assessed using a two-tailed unpaired Student’s *t* test or a two-way analysis of variance (ANOVA), with *P* values considered to be significant as follows: *P < 0.05; **P < 0.01; and ***P < 0.001. For tumor-free mice frequency, statistics were done with log-rank (Mantel–Cox) test.

### Data availability

The authors declare that all data that support the findings of this study are available within the article and its Supplementary Information files or from the corresponding author on reasonable request. RNA-seq and ChIP-seq data have been deposited into Gene Expression Omnibus (GEO) under accession GSE97831.

## Electronic supplementary material

Supplementary Information
