# Supplementary material for: BMI1 regulates androgen receptor in prostate cancer independently of the polycomb repressive complex 1
Source: Nat Commun. 2018 Feb 5;9:500. doi: 10.1038/s41467-018-02863-3 (PMC5799368; doi:10.1038/s41467-018-02863-3)
Supplement: Supplementary file 1 — Supplementary Information [file 41467_2018_2863_MOESM1_ESM.pdf]

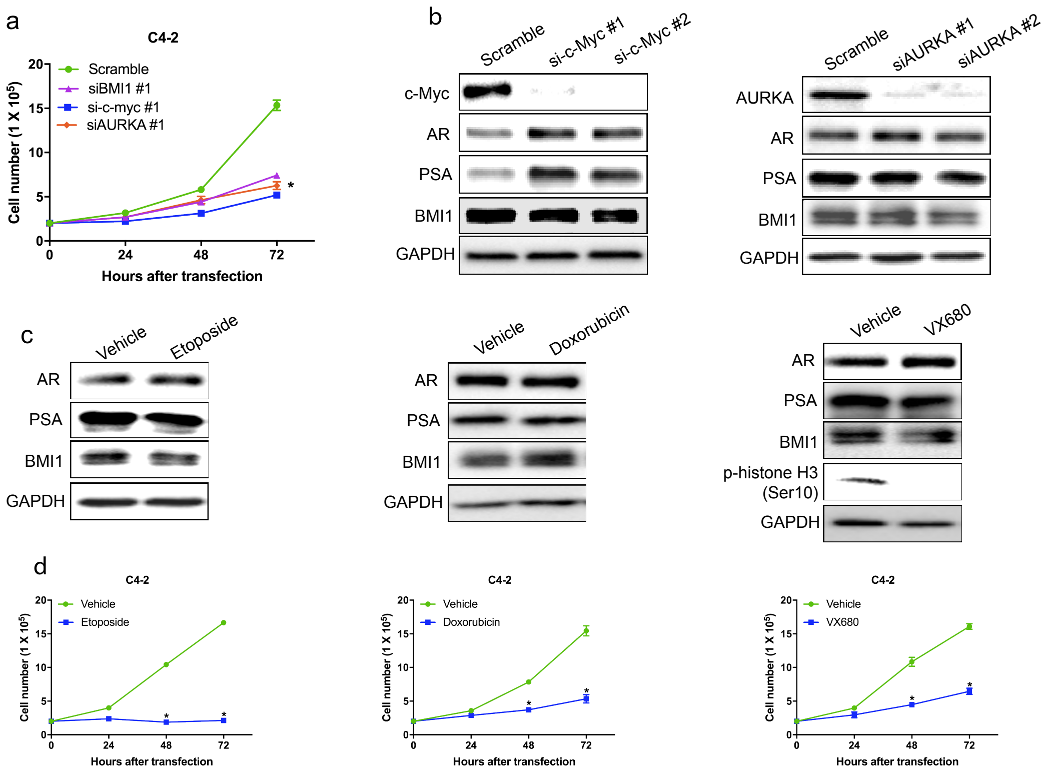


**Supplementary Figure 1 | BMI1 knockdown inhibits cell growth, and cell growth arrest has no effect on AR and PSA levels**

(**a**) $2 \times 10^5$ C4-2 cells were plated in six-well plates, grown without antibiotics for 12h, and then transfected with siBMI1, si-c-Myc, siAURKA or Scramble control, the number of cells was counted at indicated time-points. *p<0.05 vs. Scramble (Mean ± SEM, n=3). (**b**) C4-2 cells were transfected with si-c-Myc, siAURKA or Scramble control and lysed after 48hrs, followed by immunoblot analysis with anti-AR, PSA, BMI1, c-Myc, AURKA and GAPDH antibodies. (**c**) C4-2 cells were treated with Etoposide (2μM), Doxorubicin (50nM) or VX680 (5 μM) for 48h. AR, PSA, BMI1, p-histone H3(Ser10) and GAPDH were tested by western blot. (**d**) $2 \times 10^5$ C4-2 cells were plated in six-well plates, grown for 12h, then treated with Etoposide (2μM), Doxorubicin (50nM) or VX680 (5 μM), the number of cells was counted at indicated time-points. *p<0.05 vs. Scramble (Mean ± SEM, n=3)

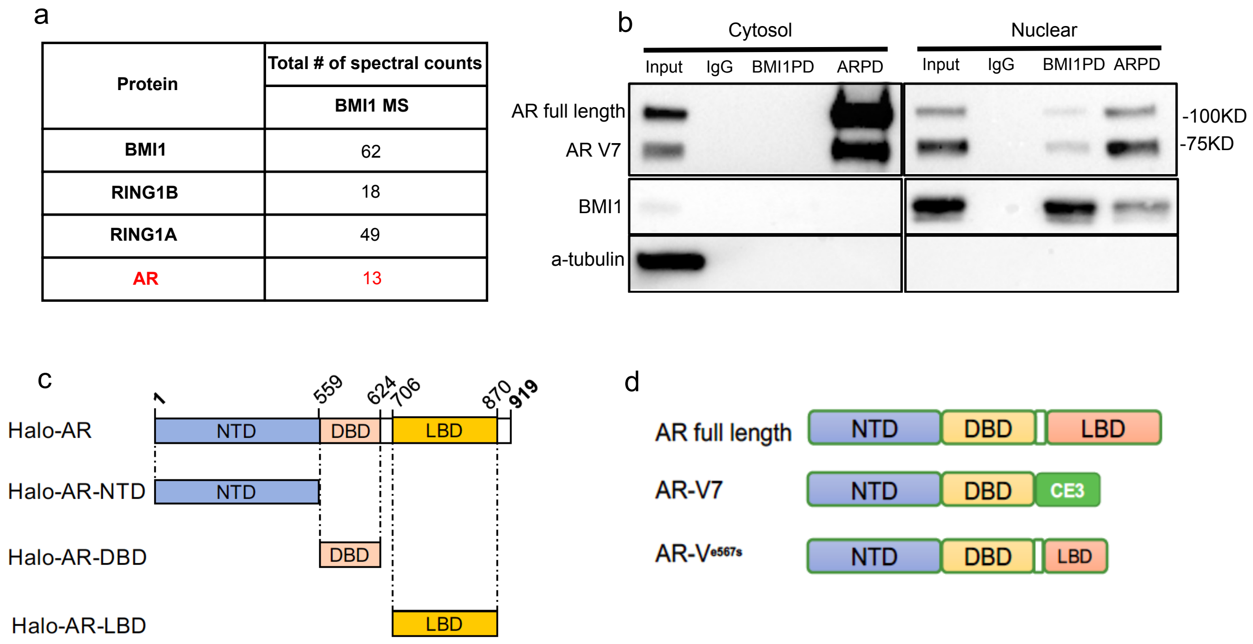


**Supplementary Figure 2 | BMI1 interacts with AR**

**(a)** Protein-protein interactions were identified by BMI1 pull-down experiment and Mass Spectrometry in VCaP cells. The number of peptides identified for each protein is indicated. **(b)** 22Rv1 cells were grown in charcoal-stripped serum for 48 hours; cytosol/nuclear fraction was subjected to pulldown assay using anti-BMI1 or anti-AR antibody; rabbit IgG used as control. **(c)** N-terminal domain (NTD), DNA binding domain (DBD), Ligand binding domain (LBD). **(d)** Domain structure of AR, cancer-associated missense mutations and splice variants: V7 and $V^{e567s}$.

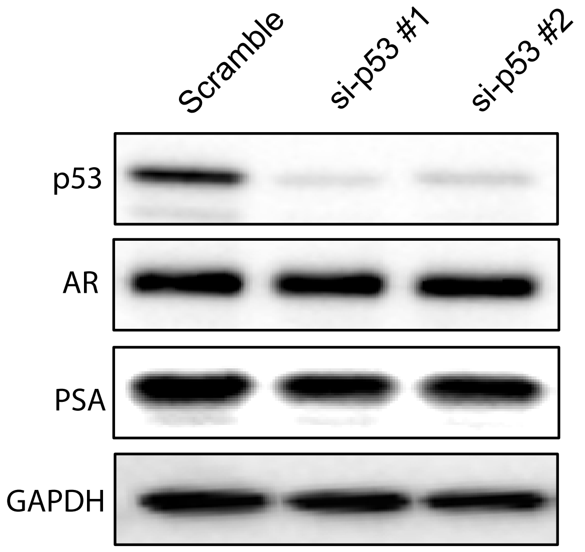


**Supplementary Figure 3 | P53 knockdown has no effect on AR signaling**

C4-2 cells were transfected with siP53 or Scramble and lysed after 48hrs, p53, AR, PSA, and GAPDH protein level was presented.

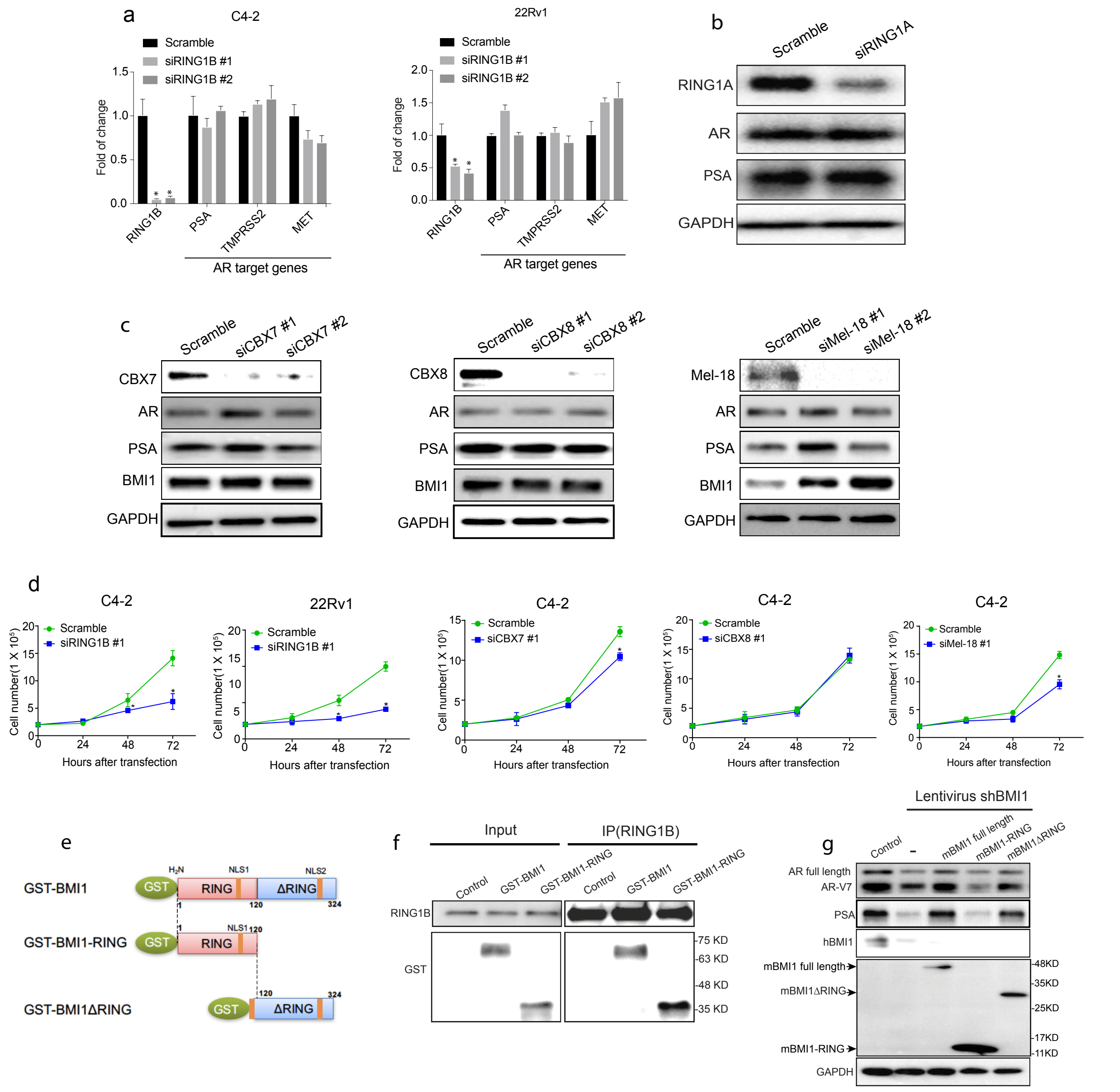


**Supplementary Figure 4 | BMI1 regulates AR signaling independently of PRC1**

**(a)** RING1B were depleted by 2 independent strands of siRNA; after 48h mRNA was extracted, RT- and quantitative PCR were performed to test AR target genes. *$p<0.05$ vs. Scramble (normalized to β-actin mRNA, Mean ± SEM, n=3). **(b)** C4-2 cells were transfected with siRING1A or Scramble and lysed after 48hrs; RING1A, AR and PSA protein level were presented. **(c)** C4-2 cells were transfected with siCBX7, siCBX8, siMel-18 or Scramble control and lysed

after 48hrs, followed by immunoblot analysis with anti-CBX7, CBX8, Mel-18, AR, PSA, BMI1 and GAPDH antibodies. **(d)** $2\times10^5$ C4-2 or 22Rv1 cells were plated in six-well plates, grown without antibiotics for 12h, and then transfected with indicated siRNA duplexes. The number of cells was counted at indicated time-points. *$p<0.05$ vs. Scramble (Mean ± SEM, n=3). **(e)** Schematic of BMI1 structures [1, 2, 3]. **(f)** GST-BMI1 or GST-BMI1-RING was incubated with RING1B for 12h followed by IPs with an anti-RING1B antibody. **(g)** 22Rv1 cells were infected with shBMI1 lentivirus, at 24hrs post infection cells were infected with flag-tagged mouse full length BMI1 lentivirus, mouse BMI1-RING lentivirus, or mouse BMI1ΔRING lentivirus as indicated; cells were lysed after another 24 hrs and probed by indicated antibodies. All experiments were biologically repeated at least three times. Representative images are shown.

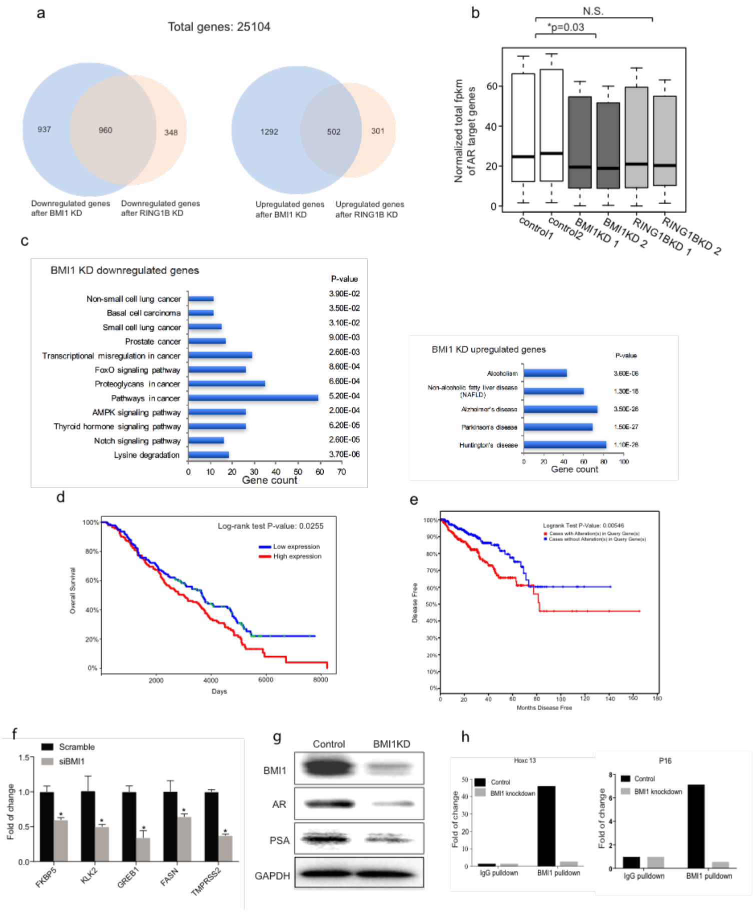


**Supplementary Figure 5 | RNA-Seq analysis shows BMI1 regulates AR independently of PRC1**

**(a)** Venn diagram shows that genes down or up regulated by BMI1 knockdown overlap with genes down or up regulated by RING1B knockdown, respectively. **(b)** Boxplot for expression level of 113 AR target genes collected from literatures (**Supplementary Table** 4). **(c)** KEGG pathways analysis shows that down regulated genes are associated with cancer pathways after BMI1 knock down. **(d)** Kaplan-Meier (KM) analysis of prostate cancer patient survival based on expression level of 53 genes that are most significantly activated by BMI1. Gene expression data were collected from the PROGgene database [4]. **(e)** Kaplan-Meier (KM) analysis of prostate cancer relapse based on mutation data of 53 genes that are most significantly activated by BMI1. TCGA

mutation data were collected from the Cbioportal database [5, 6]. **(f)** C4-2 cells were trasfected with BMI1 or control siRNA for 48 hours and then the total RNA was extracted, followed by RT- and quantitative PCR in order to measure the expression levels of AR activated genes. *$p<0.05$ vs. Scramble (normalized to β-actin, Mean ± SEM, n=3). **(g)** C4-2 were transfected with shBMI1 lentivirus or vector lentivirus, after 5 days total cell lysates were blotted for BMI1, AR and PSA, GAPDH as loading control. **(h)** BMI1 and IgG ChIP was conducted in C4-2 cells transfected with shBMI1 lentivirus or vector virus for 5 days. ChIP-qPCR was conducted using gene-specific primers.

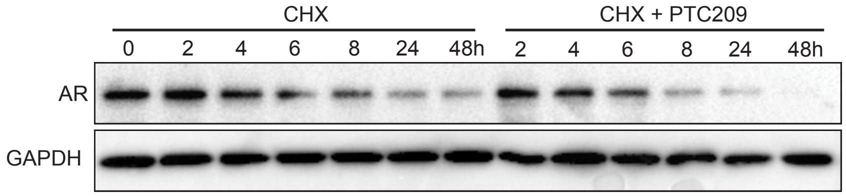


**Supplementary Figure 6 | PTC209 treatment shortens AR half-life**.

C4-2 cells were treated with PTC209 (5μM) or PTC209 (5μM) + CHX (10μg $ml^{-1}$) for the time indicated. Total cell lysates were blotted for AR, while GAPDH as loading controls.

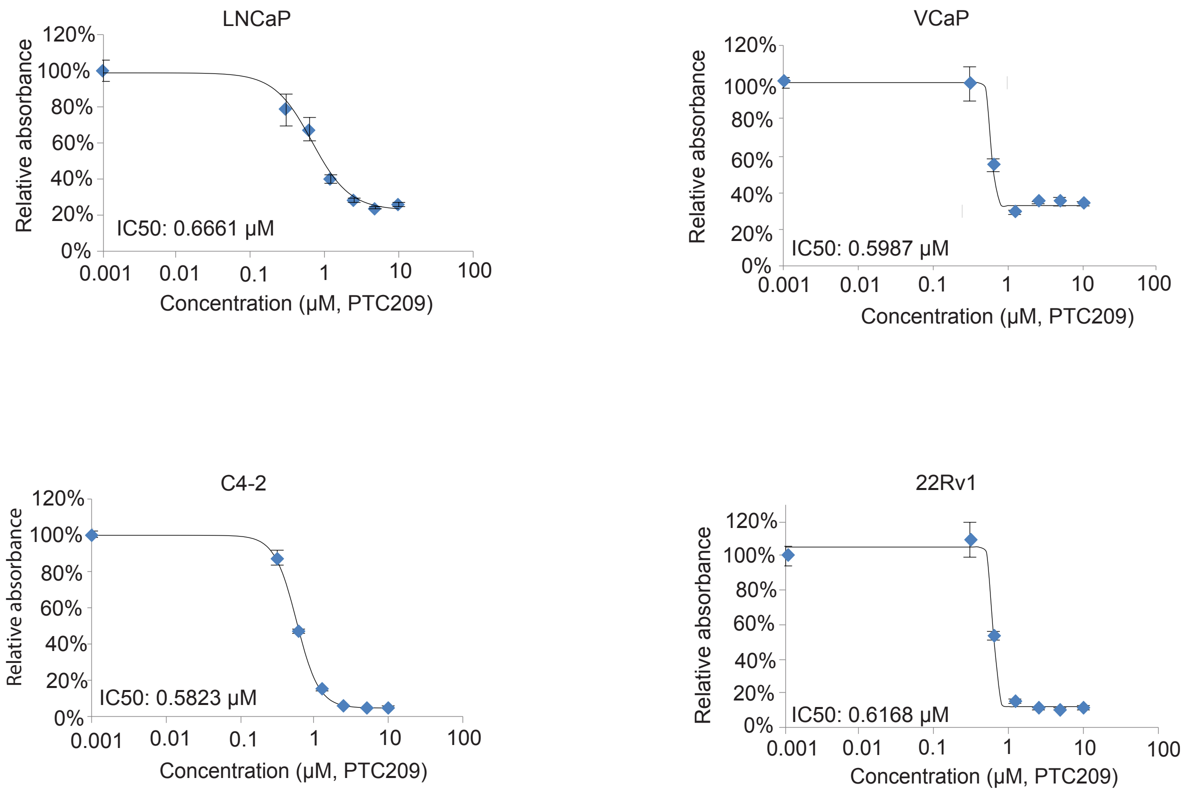


**Supplementary Figure 7 | PTC209 inhibits prostate cancer cell proliferation.**

SDA log-dose response analysis (IC50) of PTC209 in prostate cancer cell lines as indicated, Mean ± SEM.

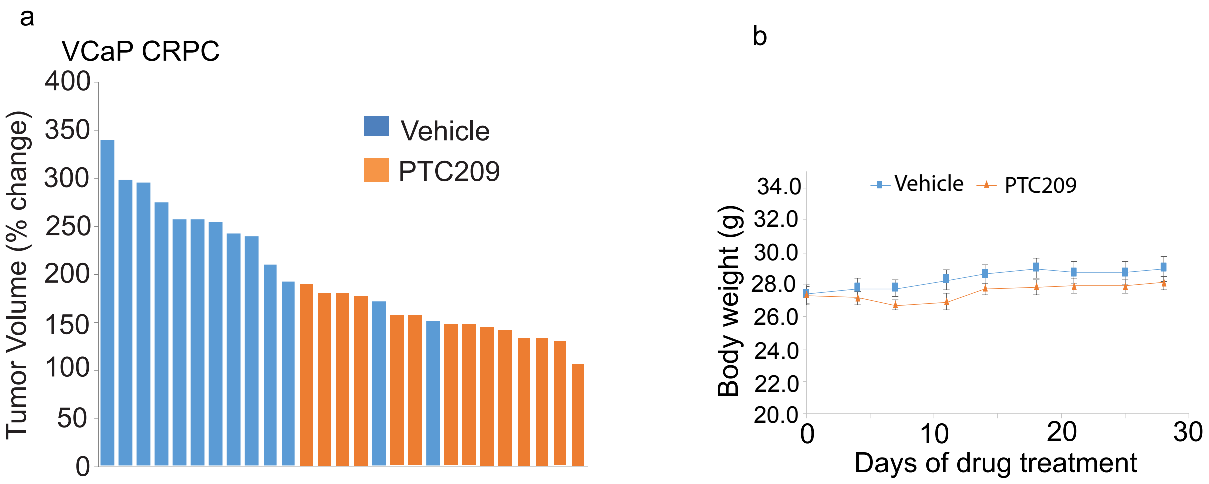


**Supplementary Figure 8 | PTC209 treatment inhibits the growth of CRPC tumors**

**(a)** Castrated mice carrying Castration-resistant VCaP xenograft received vehicle or PTC209 (60 mg $kg^{-1}$ per day) 5 days per week. Waterfall plot of tumor volume response is shown. **(b)** Body weight of mice of different groups as indicated are shown, Mean ± SEM.

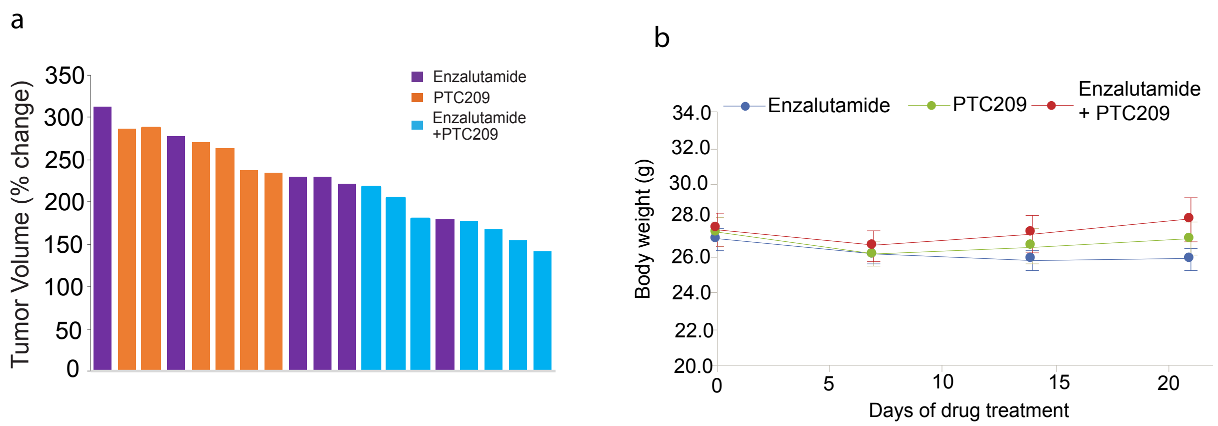


**Supplementary Figure 9 | Combinatorial treatment of PTC209 and enzalutamide shows significantly better outcomes compared to each treatment alone**.

**(a)** Castrated mice carrying CRPC xenograft received enzalutamide (10 mg $kg^{-1}$ per day), PTC209 (60 mg $kg^{-1}$ per day), or PTC209 (60 mg $kg^{-1}$ per day) + enzalutamide (10 mg $kg^{-1}$ per day) 5 days per week. Waterfall plot of tumor volume response is shown. (**b)** Body weight of mice of different groups as indicated are shown, Mean ± SEM.

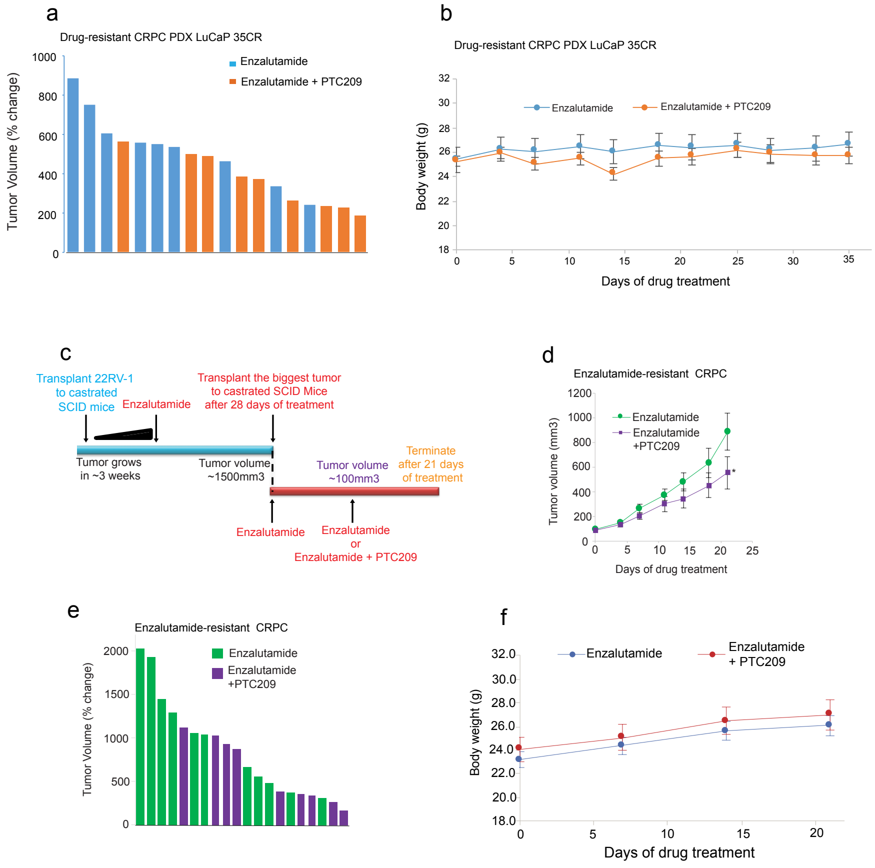


**Supplementary Figure 10 | PTC209 inhibits drug-resistant CRPC progression *In Vivo***

**(a&b)** Castrated mice carrying LuCaP 35CR received Enzalutamide (10mg $kg^{-1}$ per day) or PTC209 (60 mg $kg^{-1}$ per day) + Enzalutamide (10mg $kg^{-1}$ per day), 5 days per week (n=9 per group). Caliper measurements were taken every 4 days to obtain. **(a)** Waterfall plot of tumor volume response is shown. **(b)** Body weight of mice of different groups as indicated are shown, Mean ± SEM. **(c)** Enzalutamide-resistant CRPC mouse xenograft experimental design is illustrated. **(d-f)** Castrated mice carrying Enzalutamide-resistant CRPC xenograft received Enzalutamide (10 mg $kg^{-1}$ per day), Enzalutamide (10 mg $kg^{-1}$ per day) + PTC209 (60 mg $kg^{-1}$ per day) 5 days per week (n=9 per group); **(d)** Mean tumor volume ± SEM; **(f)** Waterfall plot of tumor volume response is shown. *P<0.05, Enzalutamide+PTC209 vs. Enzalutamide. (f) Body weight of mice of different groups as indicated are shown, Mean ± SEM.

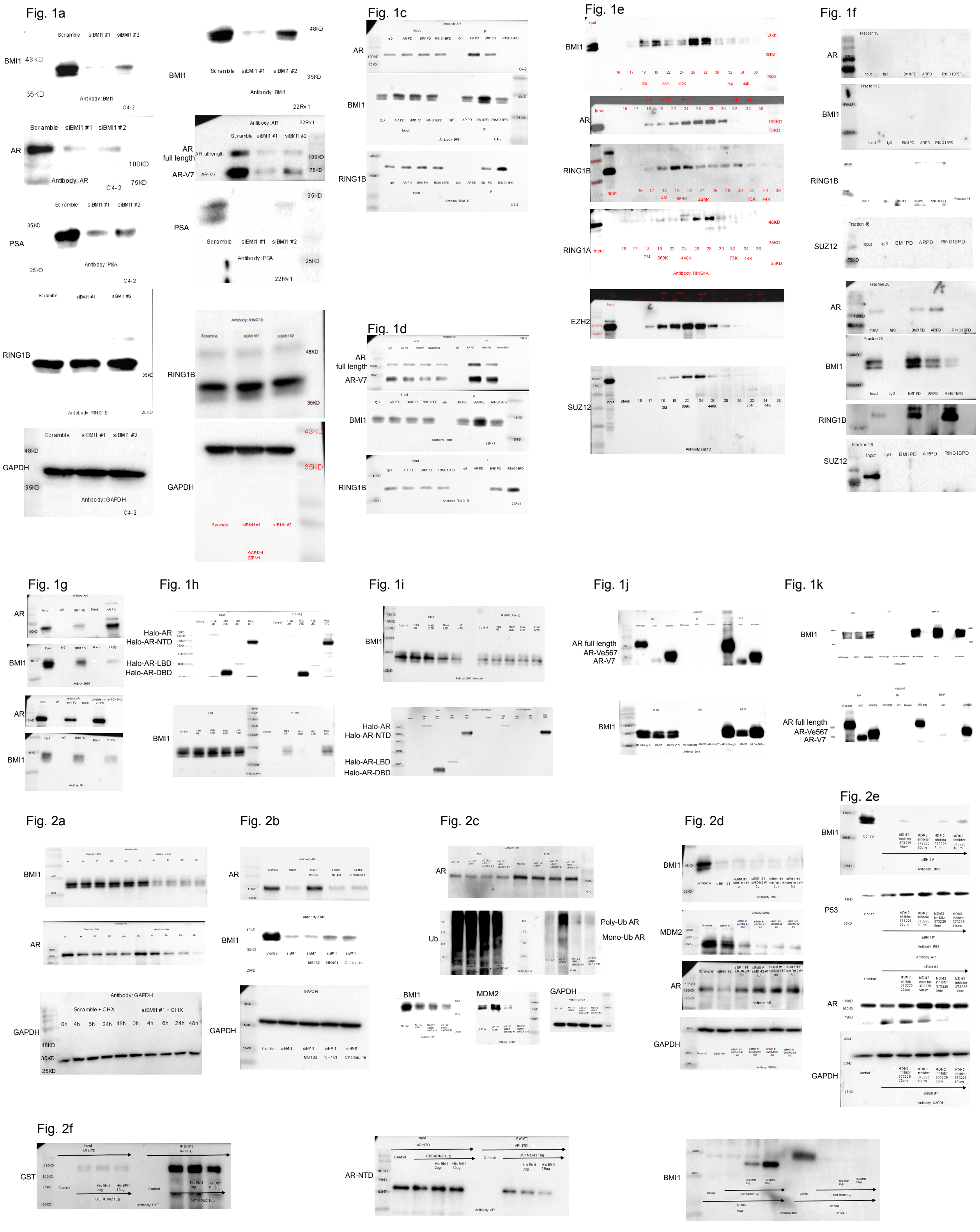


**Supplementary Figure 11 | Unprocessed blot images for the western results in Fig. 1 and 2**

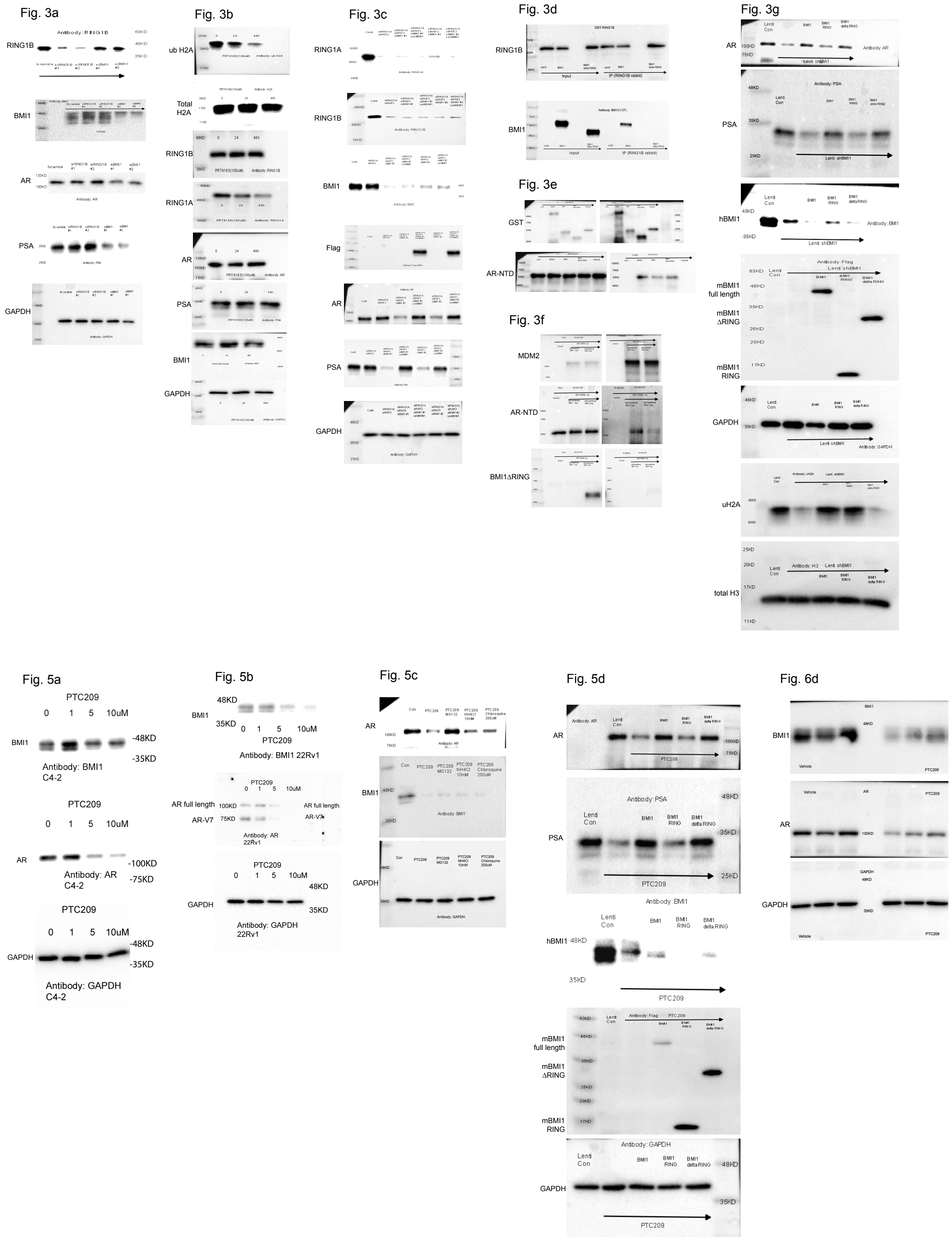


**Supplementary Figure 12 | Unprocessed blot images for the western results in Fig. 3, 5 and 6**

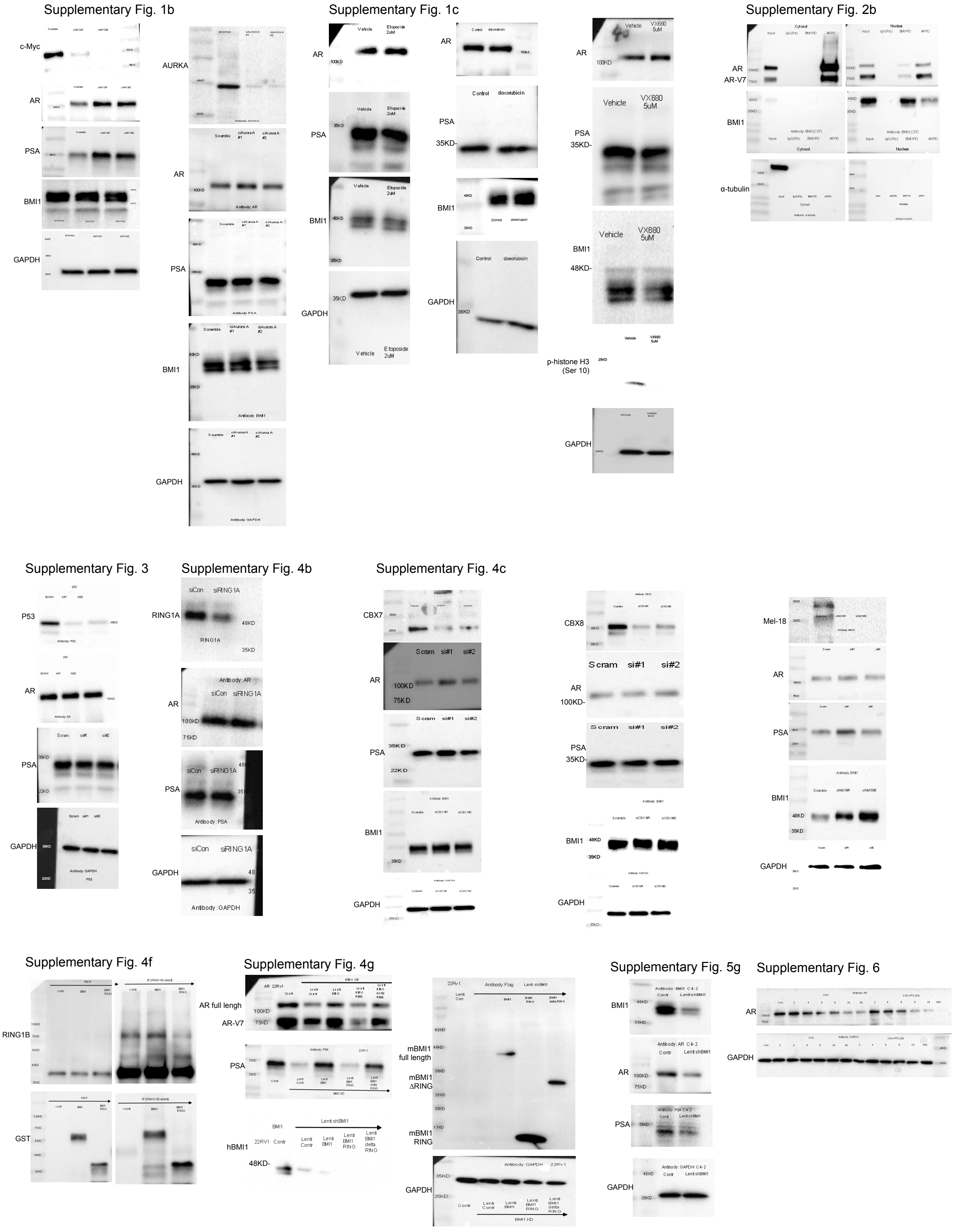


**Supplementary Figure 13 | Unprocessed blot images for the western results in Supplementary Figures**

**Supplementary Table 1 | Antibodies**

| | Host | Company | Cat# | Purpose(dilution) |
|---|---|---|---|---|
| AR | rabbit | Millipore | 06-680 | IP, Immunoblot(1:1000) |
| BMI-1(D20B7) | rabbit | Cell signaling | 6964s | IP, Immunoblot(1:1000) |
| MDM2(SMP14) | mouse | Santa Cruz | sc-965 | Immunoblot(1:1000) |
| RING1A | rabbit | Cell signaling | 2820s | Immunoblot(1:1000) |
| RING1B | rabbit | proteintech | 16031-1-AP | IP, Immunoblot(1:1000) |
| Ubiquityl-Histone H2A(K119) | rabbit | Cell signaling | 8240s | Immunoblot(1:1000) |
| Histone H3 | rabbit | Cell signaling | 9715s | Immunoblot(1:1000) |
| Histone H2A (D603A) | rabbit | Cell signaling | 12349s | Immunoblot(1:1000) |
| Prostate-Specific Antigen (PSA) | rabbit | DAKO | A0562 | Immunoblot(1:1000) |
| Ubiquitin (P4D1) | mouse | Cell signaling | 3936s | Immunoblot(1:1000) |
| GFP (B-2) | mouse | Santa Cruz | sc-9996 | Immunoblot(1:1000) |
| His-probe (H-3) | mouse | Santa Cruz | sc-8036 | Immunoblot(1:1000) |
| HA-probe (F-7) | mouse | Santa Cruz | sc-7392 | Immunoblot(1:1000) |
| GST (I-5) | rabbit | Santa Cruz | sc-459 | Immunoblot(1:1000) |
| GST (B-14) | mouse | Santa Cruz | sc-138 | Immunoblot(1:1000) |
| DYKDDDK Tag | rabbit | Cell signaling | 147935 | Immunoblot(1:1000) |
| Flag (M2.Ab) | mouse | Cell signaling | F1804 | Immunoblot(1:1000) |
| SUZ12 | rabbit | Cell signaling | 3737s | Immunoblot(1:1000) |
| EZH2 | rabbit | Cell signaling | 5246 | Immunoblot(1:1000) |
| p53 | mouse | Santa Cruz | sc-126 | Immunoblot(1:1000) |
| CBX7 | mouse | Santa Cruz | sc-376274 | Immunoblot(1:1000) |
| CBX8 | mouse | Santa Cruz | sc-374332 | Immunoblot(1:1000) |
| Mel-18 | mouse | Santa Cruz | sc-390868 | Immunoblot(1:1000) |
| c-Myc | mouse | Santa Cruz | sc-40 | Immunoblot(1:1000) |

**Supplementary Table 1 | Antibodies (Continued)**

| | **Host** | **Company** | **Cat#** | **Purpose(dilution)** |
|---|---|---|---|---|
| AURKA | rabbit | Millipore | 04-1037 | Immunoblot(1:1000) |
| p-Histone H3 (Ser 10) | rabbit | Santa Cruz | Sc-8656-R | Immunoblot(1:1000) |
| Halo tag | mouse | Promega | G921 | Immunoblot(1:1000) |
| GAPDH(6cs) | mouse | Santa Cruz | sc-32233 | Immunoblot(1:5000) |
| α-tubulin | Mouse | Cell Signaling | 3873 | Immunoblot(1:2000) |
| normal mouse IgG | | Santa Cruz | sc-2025 | IP,ChIP |
| normal rabbit IgG | | Santa Cruz | sc-2027 | IP,ChIP |
| Clean-Blot IP Detection Reagent (HRP) | | Thermo Scientific | 21230 | IP |
| Goat anti-Mouse IgG (H+L)-HRP | | GenDEPOT | SA001-500 | IP, Immunoblot(1:3000) |
| Goat anti-Rabbit IgG (H+L)-HRP | | GenDEPOT | SA002-500 | IP, Immunoblot(1:3000) |

**Supplementary Table 2 | Oligonucleotide primers for RT-PCR**

| Gene Name | Forward primer | Reverse primer |
|---|---|---|
| BMI1 | CCA TTGAATTCTTTGACCAGA A | CTGCTGGGCATCGTA AGTATC |
| AR | CAAATCACCCCCCAGGAAT | CACTGGAATAATGCTGAAGAGTAGCA |
| PSA | ACCTGCACCCGGAGAGCT | TCACGGACAGGGTGAGGAAG |
| TMPRSS2 | AGAATCGGTGTGTTCGCCTC | CTCGTTCCAGTCGTCTTGGC |
| RING1B | ATGGCACACAGAACAATGGA | ATGTGGCAACCCAAAATGAT |
| MET | CGCTGACTTCTCCACTGGTT | TACACTCCCCATTGCTCCTC |
| FKBP5 | TCTCATGTCTCCCCAGTTCC | TTCTGGCTTTCACGTCTGTG |
| KLK2 | GCTGCCCATTGCCTAAAGAAG | TGGGAAGCTGTGGCTGACA |
| GREB1 | AAGGAGGGCTGGAAACAAAT | CATTGTGGCCATTGTCATCT |
| FASN | CAGCCATGGAGGAGGTGGTGATT | CGAAGAAGGAGGCATCAAACCTA |
| β-actin | GACAGGATGCAGAAGGAGATCACT | GCGCTCAGGAGGAGCAATG |

**Supplementary Table 3 | Oligonucleotide primers for ChIP-qPCR**

| Gene Name | Forward primer | Reverse primer |
|---|---|---|
| PSA | GCCTGGATCTGAGAGAGATATCATC | ACACCTTTTTTTTTCTGGATTGTTG |
| FKBP5 | GGTTCCTGGGCAGGAGTAAG | AACGTGGATCCCACACTCTC |
| TMPRSS2 enhancer | TGGAGCTAGTGCTGCATGTC | CTGCCTTGCTGTGTGAAAAA |
| Hoxc 13 | GAGGGAACCCCAGGAGAC | CGCTCTCCACCTCTCAGC |
| P16 | GCACTCAAACACGCCTTTGC | AGAGCCAGCGTTGGCAAGGA |

**Supplementary Table 4**

| Gene Symbol | Reference | Gene Symbol | Reference | Gene Symbol | Reference |
|---|---|---|---|---|---|
| ABCC4 | [7] | GREB1 | [8] | PATZ1 | [8] |
| ABCG1 | [8] | HERC3 | [7] | PHF21A | [8] |
| ACAA1 | [8] | HMGCR | [9] | PMEPA1 | [7 8 9] |
| ACAT2 | [8] | HMGCS1 | [9] | PMS2 | [8] |
| ACOT7 | [8] | HOXC13 | [8] | POLE2 | [8] |
| ACSL3 | [7] | HPGD | [9] | PPAP2A | [8] |
| ADAM7 | [7] | IDI1 | [8] | PPP2R2A | [8] |
| AR | [9] | INSIG1 | [9] | PTGER4 | [7] |
| ATAD2 | [8] | IQGAP2 | [8] | PTPN21 | [9] |
| ATP1B1 | [8] | JARID2 | [8] | RAB3B | [9] |
| BIRC5 | [8] | KCNN2 | [8] | RFC3 | [8] |
| C1orf116 | [8] | KLK2 | [7 8 9] | RHOU | [9] |
| CAMKK2 | [8] | KLK3 | [7 8 9] | SCAP | [9] |
| CCNE1 | [8] | KLK4 | [9] | SDC4 | [8] |
| CCNG2 | [8] | LDLR | [9] | SESN1 | [8] |
| CDC25A | [8] | MAF | [7 9] | SGK1 | [9] |
| CDC6 | [8] | MAFB | [8] | SGK3 | [8] |

**Supplementary Table 4 (Continued)**

| Gene Symbol | Reference | Gene Symbol | Reference | Gene Symbol | Reference |
|---|---|---|---|---|---|
| CDKN1A | [8] | MALT1 | [8] | SI | [10] |
| CEBPD | [8] | MCCC2 | [8] | SLC45A3 | [9] |
| CENPN | [7 9] | MCM3 | [8] | SMAD1 | [8] |
| CXCR4 | [9] | MCM4 | [8] | SMPD4 | [8] |
| DBI | [9] | MCM5 | [8] | SNRK | [8] |
| DDC | [10] | MED28 | [7] | SPDEF | [8] |
| DHRS7 | [8] | MET | [10] | SQLE | [8] |
| DTL | [8] | MICAL1 | [9] | STK39 | [8] |
| EAF2 | [7 9] | MPHOSPH9 | [7] | STX3 | [8] |
| EBP | [9] | NBR1 | [8] | TAOK3 | [8] |
| EIF4A3 | [8] | NCAPD3 | [8 9] | TMPRSS2 | [7 9] |
| ELL2 | [7 8] | NDRG1 | [9] | TULP4 | [8] |
| ELOVL5 | [8] | NFAT5 | [8] | UAP1 | [8] |
| ENDOD1 | [8] | NFKB1 | [8] | VGLL4 | [8] |
| EPHA1 | [8] | NKX3-1 | [7 8 9] | WIPI1 | [9] |
| FASN | [9] | NNMT | [7] | WWC1 | [9] |
| FEN1 | [8] | ODC1 | [8] | ZBTB10 | [7] |
| FKBP5 | [7 8 9] | OPRK1 | [10] | ZBTB43 | [8] |
| FZD5 | [8 9] | ORC5 | [8] | ZFAND5 | [8] |
| GNMT | [7] | ORM1 | [9] | ZNF350 | [9] |
| GRB10 | [8] | PAK2 | [8] | | |

**Supplementary References**


1. Gray F, *et al.* BMI1 regulates PRC1 architecture and activity through homo- and hetero-oligomerization. *Nat Commun* **7**, 13343 (2016).

2. Taherbhoy AM, Huang OW, Cochran AG. BMI1-RING1B is an autoinhibited RING E3 ubiquitin ligase. *Nat Commun* **6**, 7621 (2015).

3. Bhattacharya R, Mustafi SB, Street M, Dey A, Dwivedi SK. Bmi-1: At the crossroads of physiological and pathological biology. *Genes Dis* **2**, 225-239 (2015).

4. Goswami CP, Nakshatri H. PROGgene: gene expression based survival analysis web application for multiple cancers. *J Clin Bioinforma* **3**, 22 (2013).

5. Gao J, *et al.* Integrative analysis of complex cancer genomics and clinical profiles using the cBioPortal. *Sci Signal* **6**, pl1 (2013).

6. Cerami E, *et al.* The cBio cancer genomics portal: an open platform for exploring multidimensional cancer genomics data. *Cancer Discov* **2**, 401-404 (2012).

7. Hieronymus H, *et al.* Gene expression signature-based chemical genomic prediction identifies a novel class of HSP90 pathway modulators. *Cancer Cell* **10**, 321-330 (2006).

8. Wang J, *et al.* ROR-gamma drives androgen receptor expression and represents a therapeutic target in castration-resistant prostate cancer. *Nat Med* **22**, 488-496 (2016).

9. Antonarakis ES, *et al.* AR-V7 and resistance to enzalutamide and abiraterone in prostate cancer. *N Engl J Med* **371**, 1028-1038 (2014).

10. Kim JH, *et al.* Deep sequencing reveals distinct patterns of DNA methylation in prostate cancer. *Genome Res* **21**, 1028-1041 (2011).
